# Differential effects and interactions of endogenous and horizontally acquired H-NS-like proteins in pathogenic *Escherichia coli*

**DOI:** 10.1111/j.1365-2958.2009.06995.x

**Published:** 2009-12-11

**Authors:** Claudia M Müller, György Schneider, Ulrich Dobrindt, Levente Emödy, Jörg Hacker, Bernt Eric Uhlin

**Affiliations:** 1 Department of Molecular Biology, The Laboratory for Molecular Infection Medicine Sweden (MIMS), Umeå UniversityUmeå, S-90187 Umeå, Sweden; 2 Veterinary Medical Research Institute, Hungarian Academy of SciencesBudapest, Hungary; 3 Institute of Medical Microbiology and Immunology, University of Pécs Medical SchoolPécs, H-7624 Pécs, Hungary; 4 University of Würzburg, Institute for Molecular Infection BiologyWürzburg, D-97070 Würzburg, Germany; 5 Robert Koch-InstitutBerlin, D-13353 Berlin, Germany

## Abstract

The nucleoid-associated protein H-NS is important for gene regulation in *Escherichia coli*. We have studied H-NS interaction with StpA and an uncharacterized H-NS-like protein, Hfp, in the uropathogenic *E. coli* isolate 536 that expresses all three nucleoid-associated proteins. We found distinct interactions of the three proteins at the protein level, resulting in the formation of heteromers, as well as differences in their gene expression at the transcriptional level. Mutants lacking either StpA or Hfp alone did not exhibit a phenotype at 37°C, which is consistent with a low level of expression at that temperature. Expression of the *hfp* and *stpA* genes was found to be induced by apparently diametrical conditions, and StpA and Hfp levels could be correlated to modulatory effects on the expression of different H-NS targets, the *bgl* operon and operons for virulence factors such as fimbriae and capsular polysaccharide. The *hns/hfp* and *hns/stpA* double mutants displayed severe growth defects at low and high temperatures respectively. Our findings demonstrated different requirements for the alternative H-NS/Hfp/StpA combinations under these growth conditions. We propose that Hfp and StpA have distinct functions and roles in a dynamic pool of nucleoid-associated proteins that is adapting to requirements in a particular environment.

## Introduction

The bacterial nucleoid is organized in a heterogeneous manner containing areas with highly condensed, quiescent DNA, and areas that are accessible for proteins involved in DNA processes such as replication, repair and transcription. The distribution of transcriptional activity along the chromosome is dynamic and changes rapidly according to the current requirements of the cell (Travers and Muskhelishvili, 2005). In *Escherichia coli*, several small proteins with DNA-binding activity play an important role in shaping the structure of the nucleoid and, simultaneously, in ensuring its flexibility. These so-called nucleoid-associated proteins affect the architecture of the nucleoid by either bending or bridging DNA (Dame, 2005; Luijsterburg *et al.*, 2006).

One of the most abundant nucleoid-associated proteins is the histone-like nucleoid structuring protein H-NS. The protein consists of a C-terminal DNA-binding domain and an N-terminal oligomerization domain (Ueguchi *et al.*, 1997; Renzoni *et al.*, 2001; Bloch *et al.*, 2003). The precise mechanism of DNA binding and nucleoprotein complex formation by H-NS is still unclear, despite many efforts in the last years. For instance, higher-order complexes of H-NS molecules bound along the DNA, or bridges between two adjacent DNA stretches mediated by H-NS have been shown (Rimsky *et al.*, 2001; Dame *et al.*, 2005), both with potential huge impact on DNA processes. The effects of H-NS have been studied in detail in non-pathogenic *E. coli*, where it first was suggested to cause transcriptional silencing and now is considered as a global regulator of gene expression (Göransson *et al.*, 1990; Atlung and Ingmer, 1997; Hommais *et al.*, 2001). In uropathogenic *E. coli* strain 536, we could also demonstrate a major role of H-NS on the expression of more than 500 genes, including many virulence factors such as fimbriae, cytotoxins and siderophores (Müller *et al.*, 2006).

A second member of the H-NS-like protein family that is found in both pathogenic and non-pathogenic *E. coli* variants is the StpA protein. StpA shares 58% sequence identity to H-NS and has a similar domain structure (Cusick and Belfort, 1998). It was shown to bind both DNA and RNA, the latter conferring RNA chaperone activity (Zhang *et al.*, 1996; Sonnenfield *et al.*, 2001; Mayer *et al.*, 2007). Mutations in the *stpA* gene do not result in a notable phenotype under standard growth conditions, but StpA can compensate for the lack of *hns* in repression of some genes, thereby being considered as a molecular back-up for H-NS (Sondén and Uhlin, 1996; Zhang *et al.*, 1996). Moreover, StpA is directly interacting with the H-NS protein by forming heteromers (Johansson *et al.*, 2001).

The progress in genome sequencing led to the discovery of several genes encoding homologues or interaction partners for H-NS (Bertin *et al.*, 2001; Tendeng and Bertin, 2003). Interestingly, many of those are encoded on transmissive genomic elements and many are also involved in virulence. One example is the plasmid-encoded H-NS-like protein of *Shigella flexneri*, Sfh (Beloin *et al.*, 2003; Doyle and Dorman, 2006). Sfh shares about 60% amino acid homology with H-NS and StpA and it is able to interact with both proteins (Deighan *et al.*, 2003). Furthermore, it is able to complement an *hns* mutation and affects virulence gene expression when overexpressed, but also plays an additional role in transmission and maintenance of its encoding plasmid (Beloin *et al.*, 2003; Doyle *et al.*, 2007). Acquisition of horizontally acquired proteins that are able to interact with H-NS might be an important step in fine tuning the pre-existing regulatory network of the host cell, which might explain the occurrence of several H-NS-like proteins within some bacterial species.

In this study, we provide evidence that the uropathogenic *E. coli* strain 536, but not non-pathogenic K-12 laboratory strains, harbours a third member of the H-NS-like protein family, the Hfp protein, which shares homology with both H-NS and StpA. The gene coding for Hfp is located on a genomic island inserted at the *serU* locus, and its expression is repressed by H-NS. Like StpA, the Hfp protein has biological activity and can complement several *hns* phenotypes. To study the functions and interplay of the horizontally acquired Hfp protein and the endogenous regulators H-NS and StpA, a series of mutant derivatives were constructed and searched for differences in their phenotype. Possible interactions between the three regulators were studied at the protein and the transcriptional level. The results obtained verified that a horizontally acquired regulator such as Hfp can be successfully integrated into the regulatory circuits of the cell. Moreover, our results from growth experiments and transcription analysis of the three corresponding genes led to the hypothesis of a flexible composition of the nucleoid-associated protein pool with changing temperature or with growth phase, resulting in distinct heteromeric protein complexes.

## Results

### Extraintestinal pathogenic *E. coli* variants commonly express three members of H-NS-like proteins

**Fig. 1:**
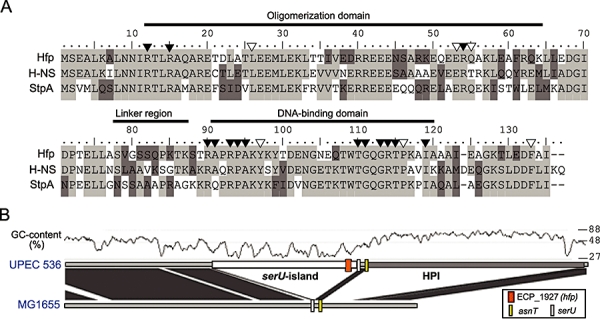
Comparison of amino acid sequences and chromosomal location of the *hfp* gene. A. Alignment of the three nucleoid-associated proteins of UPEC strain 536 (H-NS = ECP_1284; StpA = ECP_2632; Hfp = ECP_1927). Identical residues are depicted with light shading; dark shading indicates residues with > 50% similarity. The domain structure is indicated above the sequence. Residues that are essential for the function of H-NS are marked with filled arrowheads; residues that confer a dominant-negative phenotype when substituted are marked with open arrowheads [as summarized by Williams and Rimsky (1997)]. B. Genome comparison of the *hfp* region of UPEC strain 536 and non-pathogenic *E. coli* K-12 strain MG1655. Dark shading indicates regions of high homology, while horizontally acquired sequences can be identified as white regions with no homology. The localization of the *hfp* gene in the genome of the pathogen, as well as of tRNA loci is marked within the sequences. The corresponding *serU*-associated genomic island is located in close vicinity to the ‘high pathogenicity island’ (HPI) (Carniel, 2001). The GC content of the UPEC strain 536 sequence is depicted at a window size of 500 nucleotides.

The genes coding for H-NS and StpA can be found in the chromosome of all characterized *E. coli* variants. In this study, the genome of uropathogenic *E. coli* strain 536 (Accession No. CP000247), as a newly sequenced representative of extraintestinal pathogenic *E. coli* (ExPEC) isolates, was searched for the presence of H-NS-like proteins. Like *E. coli* K-12 strains, UPEC (uropathogenic *E. coli*) strain 536 possesses both the *hns* and the *stpA* equivalents. However, a third hit was obtained represented by gene ECP_1927, which is annotated as a putative DNA-binding protein (Accession No. ABG69928). The sequence encodes a protein of 134 amino acids (a.a.), which was now termed Hfp for H-NS-family protein. Hfp exhibits high homology to both H-NS and StpA (57.7% and 52.2% a.a. identity respectively) over the entire length of the sequence (Fig. 1A). When comparing the chromosomal region in the vicinity of the *hfp* gene with the corresponding region in the genome of the K-12 strain MG1655, a 23 kb large DNA stretch including the *hfp* gene was only present in the genome of UPEC strain 536 (Fig. 1B). The insertion was flanked by direct repeats associated with the *serU* tRNA locus and the overall GC content (38%) differed from the rest of the genome (50%), all of which are qualifiers for horizontally acquired DNA regions, so-called genomic islands.

In order to further investigate the occurrence of Hfp in other *E. coli* sero- and pathotypes, a collection of 133 isolates comprising both extraintestinal and intestinal pathogenic *E. coli* isolates (ExPEC and IPEC) as well as faecal isolates from healthy volunteers was screened for the presence of the *hfp* gene by PCR. A 355 bp PCR product could be amplified in 55 out of the 133 strains (Table 1). Most of the *hfp*-positive strains belonged to the ExPEC group (58% positive isolates), with UPECs as prevalent subgroup (79% positive isolates). The *hfp* gene was also present in a high number of faecal strains (39% positive isolates).

**Table 1:** Occurrence of the *hfp* gene in different *E. col i* variants.^a^

Variant	*hfp*^+^	*hfp*^-^	% *hfp*^+^
ExPEC (*n* = 67)	39	28	58.2
whereof UPEC (*n* = 42)	33	9	78.6
Faecal isolates (*n* = 39)	15	24	38.5
whereof known ECOR B2 (*n* = 11)	5	6	45.5
IPEC (*n* = 27)	1	26	3.7
Total (*n* = 133)	55	78	41.4

a The presence of the *hfp* gene was monitored in clinical isolates by PCR screening using primers Hfp-RT1 and Hfp-RT2. Extraintestinal pathogenic *E. coli* (ExPEC) included uropathogenic *E. coli* isolates (*n* = 42), strains causing sepsis (*n* = 15) and newborn meningitis (*n* = 10); intestinal pathogenic *E. coli* (IPEC) included enterotoxigenic (*n* = 4), enterohaemorrhagic (*n* = 12), enteropathogenic (*n* = 4), enteroinvasive (*n* = 2) and enteroaggregative *E. coli* (*n* = 5).

Taken together, these results indicate that UPEC strain 536, and probably a majority of other uropathogenic *E. coli* isolates, carries genes for three highly homologous nucleoid-associated proteins: the core chromosome-encoded proteins H-NS and StpA, as well as the so far uncharacterized Hfp protein.

### Hfp is directly interacting with the H-NS protein

To test whether the *hfp* gene encodes a functional gene product, plasmid pCM9 carrying the *hfp* gene and an approximately 200 nt upstream region was introduced into *hns* mutants of the K-12 strains MG1655 and MC4100, and its ability to complement for the lack of H-NS was assessed in the cases of some well-described *hns* mutant phenotypes (Fig. S1). In brief, we provide evidence that the *hfp* gene produces a functional protein which can complement for the lack of H-NS in terms of several H-NS-dependent operons.

**Fig. 2:**
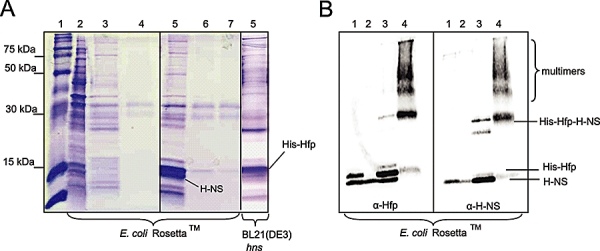
Co-purification and heteromerization of H-NS and His-tagged Hfp protein. A. Coomassie blue stain of purification fractions of His-tagged Hfp protein expressed in *E. coli* Rosetta™ (left and centre panel) and an *hns* mutant of *E. coli* BL21(DE3) (right panel) respectively. The protein was overexpressed from plasmid pCM8 and purified using Ni^2+^-affinity chromatography columns. Lanes in all panels: 1 = standard (Rainbow Marker RPN 800, GE Healthcare); 2 = column load; 3 = flow through; 4 = column wash; 5 = first fraction of eluate; 6 = second fraction of eluate; 7 = third fraction of eluate. B. Immunoblot of purification fractions from *E. coli* Rosetta™ after chemical cross-linking with 2.5 mM glutaraldehyde. Polyclonal antibodies reacting with Hfp (left panel) or H-NS (right panel), were used for detection of oligomers in cross-linked samples. Lanes in both panels: 1 = column load; 2 = flow trough; 3 = eluate before cross-linking; 4 = eluate after cross-linking. Protein bands that matched the predicted molecular weight of individual monomers, dimers and multimers are labelled accordingly. The identity of other protein bands of lower molecular weight in the crude eluate, which were also recognized by the polyclonal antiserum, remains unclear.

Heteromeric interactions between H-NS and its homologue StpA have been described (Johansson *et al.*, 2001). Evidence for a direct protein–protein interaction between H-NS and Hfp were obtained when a His-tagged version of the Hfp protein was overexpressed and purified using Ni-affinity chromatography. When the 16 kDa His-Hfp protein was eluted from the chromatography column, a second protein of approximately 15.5 kDa was co-purified in equimolar ratio (Fig. 2A). N-terminal sequencing of the smaller protein resulted in the sequence SEALKILM, which matches the H-NS sequence. Immunoblotting with antiserum recognizing H-NS confirmed its presence in all purification fractions (data not shown). When performing the same purification procedure in an *hns* mutant of the expression strain BL21, only the 16 kDa protein corresponding to His-tagged Hfp appeared in the eluate (Fig. 2A), thereby confirming that H-NS was co-purified in the first case. These results strongly indicate that Hfp, like its homologue StpA, is directly interacting with H-NS.

Next, heteromeric complexes were directly detected by chemical cross-linking of proteins in the eluate. Immunoblotting revealed the presence of a 30 kDa H-NS–Hfp dimeric complex as well as higher oligomers in the cross-linked fraction (Fig. 2B). To investigate if this heteromerization with H-NS has a stabilizing function for Hfp, as it has been shown for StpA (Johansson and Uhlin, 1999), protein stability was analysed by expressing the native Hfp protein from plasmid pCM9 in *E. coli* MC4100 derivatives lacking both StpA and H-NS, and/or the Lon protease. No degradation of Hfp in an *hns*^-^*lon*^+^ strain could be observed 3 h after stopping protein synthesis by addition of spectinomycin (Fig. S2), which suggests that the Hfp protein, in contrast to StpA, is stable even in absence of its interaction partner H-NS.

### Hfp and StpA are required for normal growth at low or high temperatures

To study the biological role of the H-NS homologues Hfp and StpA, a series of derivatives with mutations of the *hfp*, *hns* and *stpA* loci was constructed in UPEC strain 536. As a first phenotypical test, the growth ability of those strains was assessed on agar plates or in liquid medium at a temperature range between 25°C and 45°C. The data are summarized in Fig. S3 and in Table 2. The most intriguing results were obtained with the *hns/stpA* and the *hns/hfp* double mutants at the lowest and highest temperatures: in liquid medium at 45°C, the *hns/stpA* mutant exhibited a severe growth defect, with a 3.5 times longer generation time than that of the *hns/hfp* mutant (103 min versus 29 min respectively). The opposite was true at 25°C, where the *hns/hfp* mutant grew very poorly, while at this temperature, the generation time of the *hns/stpA* mutant was 1.8 times shorter in comparison with the *hns/hfp* mutant (62 min versus 111 min respectively). Taken together, our results show that *hns/stpA* and *hns/hfp* double mutants differ drastically in their growth ability at the 45°C and 25°C growth temperatures, which represents the first H-NS-independent phenotype of these mutants. This suggests that – in the absence of H-NS – functional StpA protein is required for growth at temperatures above body temperature, while Hfp promotes growth at room temperature instead.

**Table 2:** Phenotypic characterization of mutants.

Phenotype	536 wt	*stpA*	*hns*	*hfp*	*hns/stpA*	*hfp/stpA*	*hns/hfp*	*hfp/hns/stpA*
*bgl* phenotype	−	−	+	−	+	−	+	+
Motility	+	+	−	+	−	+	−	−
Haemolytic activity (30°C)	14	15	85	14	76	18	127	70
Curli fimbriae: 37°C	−	−	+	−	+	−	+	+
25°C	+	+	−	+	−	+	−	−
Autoaggregation	−	−	+	−	++	−	+++	+++
Siderophore kinetics	Slow	Slow	Fast	Slow	Fast	Slow	Fast	Fast
Generation time (min): 25°C	51	50	55	50	62	49	111	83
37°C	24	24	29	25	47	24	27	53
45°C	30	28	27	29	103	29	29	145

*Assay systems*: Generation times were assessed from cultures grown in LB medium at indicated temperatures with aeration; the *bgl* phenotype was assessed on agar plates containing the substrate X-Glu; motility was assessed on 0.3% agar plates; haemolytic activity was measured from culture supernatants and is defined as the dilution at which 50% haemolysis occurred; expression of curli fimbriae was assessed on agar plates containing congored dye; assays for autoaggregation and siderophore production were performed as described in *Experimental procedures*.

### The *stpA* and *hfp* genes are diametrically expressed with changing environmental conditions

**Fig. 3:**
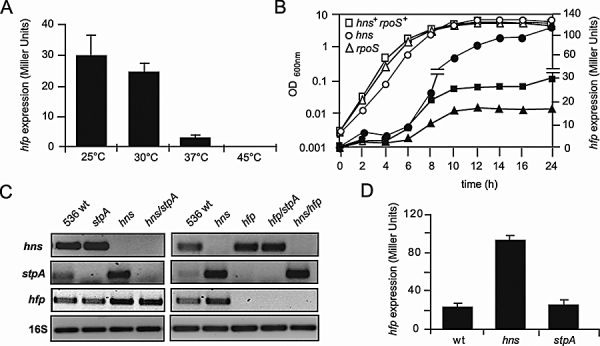
Analysis of *hfp* transcription and transcriptional cross-regulation. A. Expression of the *hfp* gene in response to temperature determined in a chromosomal 536 *hfp–lacZ* fusion strain. Bacteria were grown in LB medium at a temperature range from 25°C to 45°C. Samples for β-galactosidase measurements were taken after 16 h of incubation. Mean values and standard deviations of three independent experiments are shown. B. Expression of the *hfp* gene throughout the growth curve. The chromosomal *hfp–lacZ* fusion strain (squares) and its otherwise isogenic *hns* (circles) and *rpoS* (triangles) mutant derivatives were grown at 30°C in LB medium. Every second hour, the optical density of the culture (open symbols) and β-galactosidase activity (closed symbols) of the cultures were measured and plotted in the same graph. C. Transcript amounts of *hns*, *stpA* and *hfp* in strain 536 and isogenic mutants lacking one or two of the nucleoid-associated proteins were analysed by semi-quantitative RT-PCR using primer pairs binding in the coding sequences of *hns*, *stpA* and *hfp*. RNA samples were prepared from mid-log-phase cultures grown at 37°C. 16S rRNA was used for normalization of cDNA amounts. PCR reactions were performed in triplicate on two biological replicates per sample. The images illustrate sections of ethidium bromide-stained gels from representative experiments with each primer combination. D. Expression of the *hfp* gene as determined by measuring β-galactosidase activity from a chromosomal *hfp–lacZ* fusion. The parental strain (wt), as well as isogenic *hns* and *stpA* mutants grown at 30°C for 16 h, was analysed. Mean values and standard deviations of three independent experiments are shown.

The results derived from the growth experiments suggested distinct requirements for Hfp and StpA at low or high temperatures respectively. Therefore, it was investigated whether these differences in the requirement are reflected in the expression levels of the corresponding genes at those temperatures. When studying *hfp* expression using a chromosomal *hfp–lacZ* fusion derivative of UPEC strain 536 at a temperature range from 25°C to 45°C, a significant reduction in *hfp* expression could be observed with increasing temperature (Fig. 3A). While the *hfp* gene was clearly expressed at both 25°C and 30°C, β-galactosidase activity dropped markedly when cultures were grown at 37°C and was undetectable in cultures grown at 45°C.

The transcript levels of *hns*, *stpA* and *hfp* were also determined by reverse transcription-PCR (RT-PCR) using RNA samples from wild-type (wt) strain 536 grown at a temperature range of 25–45°C. Relatively constant levels of *hns* transcript were observed at every temperature, while *stpA* transcript levels increased with increasing temperature (data not shown), thereby indicating that the temperature-regulated expression of *stpA* described in *E. coli* K-12 (Free and Dorman, 1997) also occurs in pathogenic isolates. On the other hand, the level of *hfp* transcript decreased with increasing temperature in agreement with the *hfp–lacZ* fusion data. Moreover, we observed a clear difference in the abundance of the transcripts in the wt at 37°C when compared with each other, with *hns* being much more abundant than *stpA* or *hfp* (see also Fig. 3C, first lane). This is consistent with previous observations that *stpA* levels are usually low at 37°C (Sondén and Uhlin, 1996; Zhang *et al.*, 1996) and indicates that the same holds true for *hfp*.

The expression of *stpA* is growth phase dependent and occurs transiently during exponential growth (Free and Dorman, 1997). To analyse expression of the *hfp* gene through the batch growth phases, β-galactosidase activity from the chromosomally encoded *hfp–lacZ* fusion was measured every second hour during growth at 30°C. Only basal *hfp* expression levels could be detected throughout logarithmic phase (Fig. 3B). However, β-galactosidase activity markedly increased upon entry into stationary phase and stayed constant thereafter. The observed stationary-phase induction of *hfp* does not seem to be strictly dependent on RpoS, an alternative sigma-factor regulating many genes expressed in stationary phase. Expression of *hfp* in an *rpoS* mutant derivative of the *hfp–lacZ* fusion strain was significantly reduced (60% reduction compared with wt) but followed the same pattern of induction upon entry into stationary phase as observed in the wt (Fig. 3B). Temperature and growth phase are not the only factors that divergently regulate the expression of the H-NS homologues. Table S1 in *Supporting information* lists the differences between the expression of the *stpA* gene and the *hfp* gene as described in the literature and according to our present studies. In summary, we provide evidence that the expression levels of the H-NS-related genes *hfp* and *stpA*, which are relatively low during standard growth at 37°C, can be induced at certain, seemingly diametrical conditions, e.g. by temperature variations. Moreover, the results suggest that Hfp and StpA probably operate at temperatures other than 37°C.

### Expression of the *hfp*, *stpA* and *hns* genes is subject to negative auto- and cross-regulation

Next, the interplay between the nucleoid-associated proteins at the transcriptional level was investigated. H-NS and StpA have been shown to negatively regulate transcription of their own genes (autoregulation) as well as of their homologue (cross-regulation). To include Hfp, transcript amounts of all three nucleoid-associated proteins-encoding genes were detected by semi-quantitative RT-PCR in UPEC strain 536 and the *hfp*, *hns* and *stpA* mutant derivatives (Fig. 3C). De-repression of both *hfp* and *stpA* transcription occurred in the absence of H-NS, thereby indicating a role for H-NS as a repressor of both its homologues, in agreement with published data (Sondén and Uhlin, 1996). The negative effect of H-NS on *hfp* expression was also observed using an *hns* mutant derivative of the *hfp–lacZ* fusion strain (Fig. 3B and D). A slightly higher amount of *hns* transcript could be detected in the *stpA* mutant, as it was described before in *E. coli* K-12 (Sondén and Uhlin, 1996), but also in mutants lacking Hfp, suggesting that both StpA and Hfp negatively affect *hns* expression. A lack of StpA did not affect *hfp* transcription; vice versa, expression of *stpA* was not altered in an *hfp* mutant. To further quantify the effect of StpA on *hfp* expression, a mutation in the *stpA* gene was introduced into the *hfp–lacZ* fusion strain and β-galactosidase activity was measured from cultures grown at 30°C for 16 h (Fig. 3D). In accordance with the RT-PCR results (Fig. 3C), no alteration in *hfp* expression was observed in the *stpA* mutant background, which corroborates disparate effects of H-NS and StpA on *hfp* expression.

### The *hfp* promoter region is target for DNA binding by nucleoid-associated proteins

The repressive effect of H-NS-like proteins on target gene expression involves DNA binding to promoter regions, which also might be the mechanism for the transcriptional cross-regulation described above. To first characterize the *hfp* promoter region, various parts of the upstream region of the *hfp* gene were fused to a plasmid-encoded *lacZ* gene and transcriptional activity at different growth stages was assessed in a *lacZ* mutant derivative of strain 536 (Fig. S4A). In brief, the results indicated that transcriptional activity required upstream sequences of more than 70 nucleotides in length, with maximal activity measured with a construct encompassing 412 nucleotides upstream of the translational start codon, and confirmed both the growth phase-dependent and H-NS-regulated expression of *hfp.*

To further characterize the *hfp* promoter region, the start point of *hfp* transcription was determined by primer extension analysis. The most prominent transcriptional start point was identified at a T residue 339 bp upstream of the *hfp* translation initiation codon (see Fig. S5). Transcription from this promoter presumably is repressed by H-NS, since the amount of extension product was significantly increased in samples derived from an *hns* mutant. Analysis of the preceding sequence revealed a region of high similarity to the proposed H-NS binding motif (Lang *et al.*, 2007) between the −10 and −35 elements, which would explain the observed H-NS-mediated repression of *hfp* expression. A second transcriptional start site with an RpoS-dependent −10 box was predicted 87 bp upstream of the translation initiation codon, but the transcript was barely detectable by primer extension analyses. This proximal promoter also contained a predicted Fis site (Fig. S5), which might explain the modest degree of activation of *hfp* expression observed using *hfp–lacZ* reporter fusion derivatives (Table S1 and data not shown). Taken together, these results suggest that *hfp* is transcribed monocistronically from promoters located within a region of −70 to −412 nucleotides counting backwards from ATG and that several global regulators might be involved in regulating *hfp* expression.

**Fig. 4:**
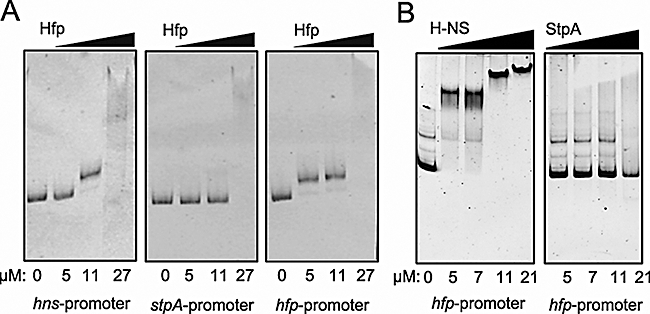
DNA-binding pattern of Hfp, H-NS and StpA to cognate promoter regions determined by electrophoretic mobility shift analysis. A. Binding activity of purified Hfp protein to PCR-generated DNA fragments spanning the promoter regions of *hns*, *stpA* and *hfp*. The figure represents ethidium bromide-stained acrylamide gels after digital inversion of the images. Protein was added at the concentrations indicated below each lane in a range from 0 to 27 µM. B. Comparison of DNA-binding affinities of H-NS and StpA at the *hfp* promoter region. The assay was carried out as described before, using the same DNA fragment as in (A). The purified proteins were added in a concentration range of 0–21 µM. Images depict representative gels of at least two independent experiments.

H-NS was shown to preferentially bind to AT-rich, intrinsically curved DNA sequences (Yamada *et al.*, 1990; Owen-Hughes *et al.*, 1992). The AT content of a 400-bp-long upstream region of *hfp* was calculated to be 63.8%, which is similar to the AT content of the corresponding *hns* and *stpA* upstream regions (60.6% and 63.5% AT respectively). A computer prediction software suggested strong intrinsic curvature of the 400 bp fragment upstream of *hfp* (data not shown). This apparent feature, and the presence of the predicted H-NS binding site, made us hypothesize that the *hfp* promoter region is a potential target for binding of nucleoid-associated proteins. To elucidate the DNA-binding activities of all three nucleoid-associated proteins to the promoter regions of *hfp*, *hns* and *stpA*, electrophoretic mobility shift assays (EMSA) were performed. Purified Hfp protein caused a minor shift of DNA fragments containing the *hfp* promoter or the *hns* promoter region, respectively, at a protein concentration of 5–11 µM, while no such shift was observed with the *stpA* promoter fragment (Fig. 4A). When studying the potential for H-NS- or StpA-mediated cross-regulation at the *hfp* promoter, H-NS was found to bind to the *hfp* fragment in a cooperative manner, resulting in extensive, stepwise shifts (Fig. 4B). No specific binding could be observed with purified StpA protein at the same conditions, thereby supporting the result of unaltered *hfp* expression in the *stpA* mutant (Fig. 3D). The DNA-binding activity of the StpA protein preparation was verified in a control experiment where binding of StpA was monitored (data not shown) to a DNA fragment representing the promoter of the *E. coli* K-12 *stpA* locus (Sondén and Uhlin, 1996). In summary, our results with DNA representing the *hfp* and *hns* promoter regions suggested that Hfp has a recognizable albeit weak DNA-binding activity. Furthermore, the difference in the shifts caused by H-NS and Hfp at a given protein concentration indicated that there would be differences in the arrangement or the stability of the DNA–protein complexes formed by the two proteins.

### Differential modulation of gene expression by Hfp and StpA

The data obtained from transcriptional analysis suggested that the effects of Hfp and StpA are the strongest at temperatures other than 37°C. This was also confirmed by the observation that both the *hfp* and the *stpA* single mutants exhibited no obvious phenotype when tested for the expression of the known major virulence traits of strain 536 at 37°C (Table 2). Lack of H-NS on the other hand affected the expression of all factors tested, resulting in a hyper-fimbriated, hyper-haemolytic, but non-motile phenotype at 37°C, as described before (Müller *et al.*, 2006). The phenotypes at 37°C of the double and triple mutants were solely dependent upon H-NS but not of its homologues StpA or Hfp at that temperature.

**Fig. 5:**
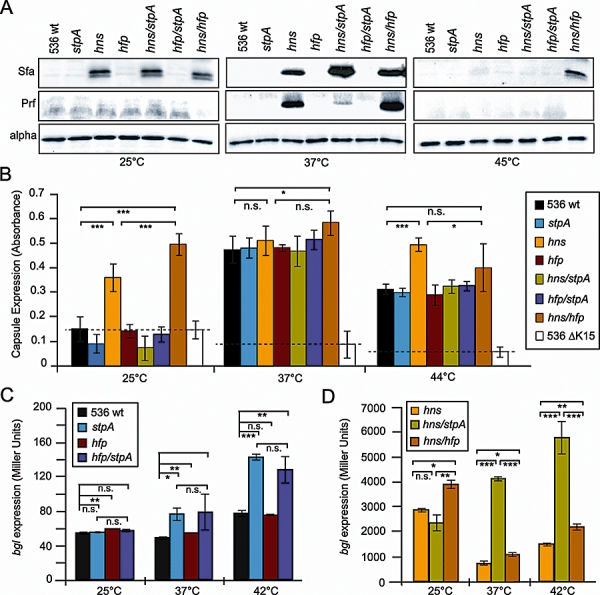
Effect of Hfp and StpA on gene expression. A. Effect of the lack of nucleoid-associated proteins on fimbriation. The protein levels of the major subunits of S and P fimbriae (SfaA and PrfA respectively) were determined by Western blotting from cultures of UPEC strain 536 and mutants lacking one or two of the nucleoid-associated proteins H-NS, Hfp and StpA. All strains were grown to mid-log phase at 25°C, 37°C and 45°C respectively. The alpha-subunit of the RNA polymerase was used as a control for equal loading of whole cell lysates. Images depict representative blots of at least two independent experiments. B. Effect of the H-NS homologues on the production of capsular polysaccharide on the cell surface. Strain 536 and the series of isogenic *hns*, *stpA* and *hfp* mutant derivatives were grown overnight at 25°C, 37°C and 44°C, and cultures were adjusted to the same optical density. The K15 capsule was detected by ELISA using pre-absorbed K15 capsule-specific antiserum and HRP-conjugated secondary antibodies and quantified by measuring the optical density at 490 nm. Mean values and standard deviations from three independent experiments are shown. C and D. Effect of the H-NS homologues on expression from the H-NS-regulated *bgl* promoter. Transcriptional activity was measured in strain 536 and isogenic mutants lacking *stpA* and/or *hfp* (C) and the series of *hns* mutant derivatives (D). In both (C) and (D), all strains carried a transcriptional *bgl–lacZ* fusion on a plasmid (pKEN61). The strains were grown to mid-log phase at 25°C, 37°C and 42°C, as indicated in the figure. Mean values and standard deviations from three independent experiments are shown. In (B–D): Values marked with one asterisk are statistically significant with a *P*-value of < 0.05, two asterisks indicate a *P*-value < 0.01, and three asterisks indicate a *P*-value < 0.001 as calculated using the Student's *t*-test.

Fimbrial adhesins are crucial factors for virulence of uropathogenic *E. coli*. When comparing expression of two of the fimbrial operons of UPEC strain 536 in the *hns/stpA* and the *hns/hfp* mutant, a clear difference was observed: as determined from the immunoblot analyses of samples from 37°C, lack of StpA resulted in a less pronounced de-repression of Prf-pili in the absence of H-NS, whereas expression of Sfa-pili was even higher compared with the *hns* single and *hns/hfp* double mutants (Fig. 5A; middle panel), which indicates differences in the ability of StpA and Hfp to modulate fimbriation at 37°C. The expression of fimbriae was also tested at 25°C and 45°C (Fig. 5A; left and right panel respectively). A de-repression of the Sfa-pili in strains lacking H-NS could be observed at 25°C, as described for 37°C (Fig. 5A; middle panel), while at 45°C Sfa-pili were only produced by the *hns/hfp* double mutant, which indicates that StpA alone was not able to repress *sfa* expression at this temperature. Prf-pili could not be detected in any of the strains at 25°C and 45°C. This seems consistent with a tight regulation of the expression of those virulence traits that would be optimized to the conditions in a mammalian host, and also suggests that StpA and Hfp probably do not play any major role in regulation of virulence factor expression in that specific environmental setting.

The expression of the capsule determinant was investigated as a second potential target operon, since H-NS was shown to affect the expression of the region 1 genes of the K5 capsule in a temperature-dependent manner by repressing transcription at 25°C, but being required for maximum expression at 37°C (Corbett *et al.*, 2007). The role of the nucleoid-associated proteins in the production of capsular polysaccharide in UPEC strain 536 was assessed by ELISA. In 536 wt bacteria, the highest expression level of capsular polysaccharide was detected at 37°C, in agreement with published data (Corbett *et al.*, 2007), whereas the expression level dropped to 60% at 44°C, and only background levels were reached at 25°C (Fig. 5B). When the mutant series was analysed, only the *hns* and *hns/hfp* double mutants (but not the *hns/stpA* mutant) showed alterations in capsule expression. At 25°C, reactivity with the K15 antiserum was 3.1-fold and 4.5-fold increased in the *hns* and *hns/hfp* mutants, respectively, as compared with wt, which also coincided with a mucoid phenotype of these mutants at this temperature on plates. At 37°C, all mutants behaved like the wt, except the *hns/hfp* mutant that showed a slightly but significantly higher capsule production. At 44°C, only the *hns* mutant yielded significantly higher values as compared with wt. These results confirm a role of nucleoid-associated proteins in the production of the capsular polysaccharide of serotype K15 in a temperature-dependent manner.

To further characterize the roles of Hfp and StpA in transcription, a reporter system for *bgl* expression was used as a well-described target for regulation by H-NS-like proteins. A plasmid containing a transcriptional *bglG::lacZ* fusion (pKENV61) was introduced into strain 536 and the set of mutants and β-galactosidase activity was measured from cultures grown at 25°C, 37°C or 42°C (Fig. 5C and D). Since the strains used are all *lac*^+^ on the chromosome, a control experiment was performed using plasmid pKENV61Δ, where a part of the *bglG::lacZ* fusion was deleted. In all strains and conditions tested, the background β-galactosidase activity measured was less than 20 Miller units (data not shown). When measuring β-galactosidase activity in strains carrying pKENV61, all strains lacking H-NS exhibited de-repressed *bgl* transcription at any of the temperatures tested, with the strongest effect observed at 25°C (Fig. 5C and D). Interestingly, *bgl* expression was four to five times higher in the *hns/stpA* double mutant compared with the *hns* single mutant at 37°C and 42°C, but not at 25°C (Fig. 5D). The same effect was observed in an *hns*^+^ background, where de-repression in both *stpA* and *hfp/stpA* mutants compared with the wt increased with increasing temperatures (Fig. 5C). These results suggest that StpA has an additive effect in the repression of the *bgl* operon and that the strength of repression correlates with the expression levels of the *stpA* gene, i.e. high StpA levels at 42°C coincide with a strong repressive effect on *bgl* transcription. Lack of Hfp alone did not result in de-repression of the *bgl* operon (Fig. 5C). However, lack of both H-NS and Hfp resulted in higher β-galactosidase values than those obtained by the lack of H-NS alone, and this additional increase in *bgl* expression was the highest at 25°C, where the difference between the *hns/hfp* and the *hns* single mutant was more than 1000 Miller units (Fig. 5D). From this, we can conclude that Hfp, in contrast to StpA, does not contribute to repression of the *bgl* operon when H-NS is present. In the absence of H-NS, however, Hfp can exert a repressive effect that is the strongest at low temperatures, two conditions where Hfp levels are induced.

Taken together, as exemplified by the *bgl* operon and the capsule determinant, a clear correlation could be made between *hfp* and *stpA* transcript levels and the strength of modulatory effects of the encoded proteins on the expression of H-NS-regulated genes. This suggests that the H-NS homologues Hfp and StpA can act as modulators of gene expression at divergent environmental conditions.

## Discussion

**Fig. 6:**
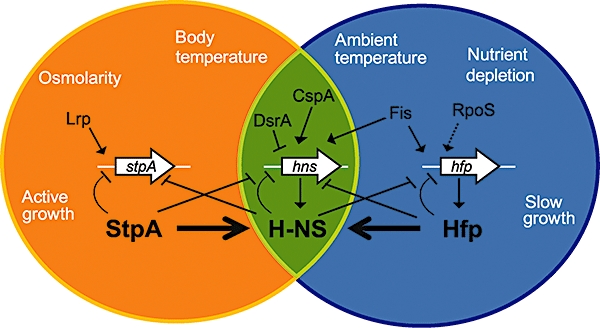
Model for occupation of disparate niches by the H-NS homologues StpA and Hfp. In contrast to the abundant H-NS protein that is produced in various types of environment, expression of the *stpA* and *hfp* genes is induced by disparate environmental conditions mediated through differences in transcriptional regulation. Interactions between the H-NS-like proteins include modulating effects of both StpA and Hfp on H-NS function by heteromerization and effects on *hns* expression through a complex network of transcriptional cross-regulation.

In this study, we provide evidence that many ExPEC, as exemplified by UPEC strain 536, encode three members of the H-NS-like protein family, the global regulator H-NS itself, as well as two intraspecies homologues StpA and Hfp, similar to the situation described for the StpA and Sfh proteins of *Shigella* (Beloin *et al.*, 2003). We suggest that the so far uncharacterized *hfp* gene and its gene product are not to be considered simply the redundant result of gene duplication, but that Hfp represents a distinct functional member in a homeostatically regulated pool of H-NS-like proteins. In this respect, our findings support existing hypotheses (Dorman, 2004). This protein pool includes full-length H-NS homologues such as StpA or Hfp, and, as shown by Juarez and co-workers (Madrid *et al.*, 2007), also more distant homologues like members of the Hha/YmoA family. In particular, our study demonstrates that the composition of the pool of nucleoid-associated proteins in *E. coli* 536 is distinctly regulated and changes according to environmental conditions, resulting in variable levels of Hfp or StpA as interaction partners for the abundant H-NS protein. The latter suggestion is derived from our transcription studies of the *hfp* and *stpA* genes. The expression levels of both genes are relatively low under standard laboratory growth conditions as described here and elsewhere (Sondén and Uhlin, 1996), which might account for the apparent lack of phenotype of *hfp*, *stpA* or *hfp/stpA* double mutants at 37°C. Presumably, it indicates that the major effects of Hfp or StpA are not exerted at this temperature. On the other hand, as summarized in Table S1, expression of the two H-NS homologues can be markedly increased under certain conditions, e.g. at lower temperature and upon entry into the stationary growth phase for *hfp* expression, or at higher temperatures (> 40°C) during active growth for *stpA* expression. The inducing conditions for elevated Hfp and StpA levels thereby seem to be quite diametrical, suggesting that Hfp and StpA rarely encounter each other in the cell. Therefore, we propose a scenario in which Hfp and StpA have evolved to being divergently regulated through selective forces exerted by different environmental niches (see Fig. 6).

Despite the differences in expression levels, the biochemical parameters of the histone-like proteins are very similar. Like StpA, the Hfp protein exhibits high homology to H-NS, thereby also suggesting a similar two-domain structure. Homology between Hfp and H-NS is the highest in the region corresponding to the N-terminal oligomerization domain, which might account for the observed heteromerization between Hfp and H-NS. In contrast to StpA, which has been reported to be readily degraded by the Lon protease if not protected by complex formation with H-NS (Johansson and Uhlin, 1999), heteromerization with H-NS did not seem to be required for stability of Hfp, which demonstrates distinct properties of the Hfp and StpA proteins. Heteromerization with H-NS might be an important aspect in the role of Hfp and StpA in the cell. Due to the observed differences between H-NS and Hfp in their interaction with DNA (Fig. 4), we need to consider the possibility that a heteromeric complex might exhibit yet another distinct DNA binding property. Two examples of promoters that are regulated by alternating nucleoprotein complexes is the region 1 promoter of the K5 capsule determinant and the promoter of the H-NS-silenced gene for the cryptic haemolysin HlyE, also known as ClyA (Westermark *et al.*, 2000; Corbett *et al.*, 2007; Lithgow *et al.*, 2007). In these cases, both H-NS and the SlyA protein bind to overlapping sites within the promoter region, resulting in higher-order protein : DNA complexes, depending on the relative concentrations of the DNA binding proteins. Our results using strain 536 suggest that H-NS represses the production of the K15 capsule at low temperatures, as it was described for other group 2 capsule determinants (Corbett *et al.*, 2007). In contrast to the K5 capsule, H-NS seems not to be required for maximal expression of the K15 capsule at 37°C, since no reduction was observed in the *hns* mutant compared with the wt. At lower temperatures, on the other hand, Hfp has a clear repressive effect on the K15 determinant, since the level of de-repression in the *hns/hfp* mutant differed significantly from that of the *hns* mutant at 25°C (Fig. 5B). As determined by EMSA, both purified H-NS and Hfp proteins were able to bind to a DNA fragment spanning the region 1 promoter (our unpublished data). Therefore, a participation of Hfp in the nucleoprotein complex formed at the capsule determinant might occur.

Differential effects of Hfp and StpA were observed for other operons as well, such as the fimbrial and *bgl* determinants. Nevertheless, most of the effects of Hfp and StpA on gene expression could be revealed when H-NS was absent, i.e. in the *hns/hfp* and *hns/stpA* double mutants. This might partly result from the low expression levels of *stpA* and *hfp* in the wt due to repression by H-NS, thus rendering H-NS much more abundant, which might mask the effects of the H-NS homologues. In the absence of H-NS, on the other hand, Hfp and StpA levels increase and they can assume some of the H-NS functions. However, the results obtained from our growth studies clearly indicate that Hfp and StpA are not simple ‘molecular backups’ for H-NS.

Characterization of the H-NS-like genes and proteins also involves questions about what selective forces may promote establishment of genes moved by lateral gene transfer. In this study, we also investigated the presence of the *hfp* gene and the *serU*-associated genomic island by PCR screening of a large set of different *E. coli* pathotypes and variants. The *hfp* gene was mainly detected in extraintestinal pathogenic isolates, but is also present in some faecal isolates. Since the faecal flora is considered to be the reservoir for uropathogenic *E. coli*, and many of the faecal isolates that carried the *hfp* gene belonged to ECOR group B2, the phylogenetic lineage that comprises mostly extraintestinal pathogenic isolates, the distribution pattern of *hfp* among faecal ECOR B2 strains observed can be explained. It is also very striking that only one out of 27 IPEC strains included in our PCR screen, EAEC isolate O42, was positive for the *hfp* gene. However, both uropathogenic and faecal isolates were over-represented in the strain collection compared with IPEC strains, which could result in a certain bias. Therefore, it remains to be elucidated whether there is a specific selection for the *hfp* gene in non-IPEC variants. Moreover, amplification of a PCR product of the right size does not imply an unaltered nucleotide sequence or a functional protein. Hence, not all *hfp*-positive strains can be necessarily considered to express the *hfp* gene.

In conclusion, many pathogenic *E. coli* isolates encode two distinct H-NS homologues, Hfp and StpA, which differ in their function and in the expression levels of the respective genes. All three proteins exhibit distinct interactions at the transcriptional and at the protein level. These interactions result in fine-tuned expression of a broad subset of target genes, including virulence-associated genes such as fimbriae genes and the capsular polysaccharide genes in UPEC strain 536. Moreover, Hfp exemplifies a regulatory protein encoded on a genetic element acquired by lateral gene exchange which made an impact on the regulatory circuits of the cell as an integrated partner in the pool of nucleoid-associated proteins.

## Experimental procedures

### Bacterial strains and culture conditions

All strains and plasmids used in this study are listed in Table S2. The strain collection used for the PCR screening has been described elsewhere (Dobrindt *et al.*, 2001). Unless stated otherwise, bacteria were grown with aeration in Luria broth (LB) at 37°C. The temperature 44°C or 45°C was used as high temperature condition, except when using plasmid containing strains, since the *hns/hfp* and *hns/stpA* double mutants were unable to grow at 45°C in the presence of antibiotics in the growth medium. For the detection of siderophores, cultures were grown overnight in MM9 medium (Schwyn and Neilands, 1987) and iron-limited conditions were induced during 3 h by adding 0.1 mM of the chelator 2,2′-dipyridyl. When required, antibiotics carbenicillin and kanamycin were used at concentrations of 100 µg ml^−1^ and 30 µg ml^−1^ respectively.

### Mutagenesis

The construction of deletion mutants was performed as described earlier (Müller *et al.*, 2006) using the λ-Red mutagenesis method (Datsenko and Wanner, 2000). The chromosomal *hfp–lacZ* transcriptional fusion strain was constructed in a similar approach: a 6.5 kb linear DNA fragment containing a promoterless *lacZ* gene followed by *lacY*, *lacA* and the *neo* gene, promoting kanamycin resistance, was PCR amplified with primers Hfp–lacZ1 and Hfp–lacZ2, using plasmid pVIK112 as template. The primers contained 54 nt and 48 nt homology extensions that allowed λ-Red recombinase-mediated recombination into the intergenic region between the *hfp* gene and the downstream ORF ECP_1928, thus leaving an intact copy of the *hfp* gene on the chromosome. Bacterial clones with successful integration were selected on agar plates containing 5-bromo-4-chloro-3-indoyl-β-d-galactopyranoside (X-gal) and kanamycin. All mutations were confirmed by both PCR and Southern hybridization.

### Plasmid construction

For overexpression of His-tagged Hfp protein, the *hfp* sequence was amplified from the chromosome of UPEC strain 536 by using primers XhoI-His-Hfp and Hfp-HindIII, which contained a hexa-His sequence, a factor XA cleavage site, as well as XhoI or HindIII restriction sites. After enzymatic digest, the fragment was ligated into the XhoI/HindIII sites of the expression plasmid pASK75, resulting in plasmid pCM8. For complementation experiments, the native *hfp* gene with its own promoter was amplified by PCR using primers Hfp-fw-HindIII and Hfp-rv-XbaI, and the 750 bp fragment was cloned into the HindIII/XbaI sites of the low-copy-number plasmid pWKS30, resulting in plasmid pCM9. The promoter fusion constructs were created by PCR amplification of different parts of the upstream region of the *hfp* gene using the Hfp-EcoRI series of forward primers in combination with the reverse primer Hfp-BamHI binding immediately after the translational start codon of the *hfp* sequence. The PCR products were cloned into the EcoRI/BamHI sites of plasmid pRZ5202, resulting in plasmids pCM15-pCM19. All inserts were sequenced for verification.

### RNA isolation and RT-PCR analysis

Total RNA extraction and semi-quantitative RT-PCR were performed as described previously (Müller *et al.*, 2006). The sequences of the oligonucleotides used for RT-PCR are listed in Table S3 in *Supporting information*.

### Phenotypic tests

The utilization of beta-glucosides (*bgl* phenotype) was detected on agar plates containing indicator dye and 40 µg ml^−1^ 5-Bromo-4-chloro-3-indolyl-d-glucopyranoside (X-Glu; Sigma) as substrate, where white colonies were scored as *bgl*-negative, and blue colonies as *bgl*-positive (Johansson *et al.*, 1998). Motility, haemolytic activity and the production of siderophores were assessed as described before (Müller *et al.*, 2006). Production of curli adhesin was detected after 3 days of incubation on no-salt LB plates supplemented with 40 mg l^−1^ congored dye and 20 mg l^−1^ Coomassie Brilliant Blue R-250. Results were scored as + = red colonies or − = white colonies. Auto-aggregation was assessed at room temperature in M63BI medium after adjusting optical densities of the cultures as described elsewhere (Roux *et al.*, 2005) and reported here as absent (−), slow (+) and fast (++).

### β-Galactosidase assay

The β-galactosidase activity was determined as described by Miller (1972). Experiments were performed in triplicate from independent cultures, with three separate measurements for each sample.

### Purification of recombinant His-Hfp protein

*Escherichia coli* strain BL21 (DE3) Δ*hns* was used for overexpression of recombinant Hfp protein from plasmid pCM8. Approximately 800 ml of LB cultures were grown at 30°C to OD_600_ = 0.2, before adding the inducer anhydro-tetracycline at a final concentration of 0.2 µg ml^−1^. Protein synthesis was carried out for 6 h. Cells were pelleted by centrifugation and resuspended in 10 ml of LEW buffer (50 mM NaH_2_PO_4_, 300 mM NaCl, pH 8) containing protease inhibitor cocktail (Complete Mini, Roche). Cells were lysed by sonication for 10 × 20 s pulses with 20 s cooling periods in between. After two rounds of centrifugation at 18 000 *g* for 20 min at 4°C, the cleared lysate was loaded onto Ni^2+^− columns (Protino Ni1000, Macherey-Nagel). Affinity chromatography was performed following the manufacturer's recommendations. Eluted protein was dialysed into storage buffer [150 mM NaCl, 50 mM Tris-HCl (pH 8), 1 mM EDTA, 0.5 mM DTT, 20% glycerol] and concentrated using Vivaspin 10 000 columns (Vivascience). The His-tag was removed by factor XA digest (New England Biolabs) overnight at room temperature. For final purification by gel filtration, 1 ml of protein samples after factor XA treatment were loaded onto a HiLoad Superdex 75 prep grade column (Amersham Biosciences) and eluted with a buffer containing 150 mM NaCl, 50 mM Tris-HCl (pH 7.5) in 2 ml fractions.

### *In vivo* protein stability assay

Stability of native Hfp protein expressed from pCM9 was tested as described by Johansson and Uhlin, (1999). Briefly, 20 ml of cultures were grown to OD_600_ = 0.4, before inhibiting protein synthesis by the addition of 100 µg ml^−1^ spectinomycin. Samples for Western blotting were taken before and 180 min after the addition of spectinomycin.

### Electrophoretic mobility shift assay (EMSA)

DNA–protein interactions were studied by a gel retardation assay using pure H-NS and StpA proteins or recombinant Hfp protein purified from *E. coli* BL21 Δ*hns* as described above. As substrate for binding, 400 bp PCR products containing the promoter regions of *hfp*, *hns* and *stpA* were used. The composition of the reaction buffer was: 25 mM HEPES (pH 7.3), 0.1 mM EDTA, 5 mM DTT, 10% glycerol, 50 mM KCl and 0.1 µg of poly(dIdC) non-specific competitor DNA. Purified protein (0–27 µM) was incubated with 100 ng of PCR products for 20 min at room temperature. Samples were loaded onto 6% Tris-glycine gels and DNA was visualized by ethidium bromide staining.

### Chemical cross-linking

Interactions between Hfp and H-NS were studied by chemical cross-linking for 20 min at room temperature using 2.5 mM glutaraldehyde as described previously (Lindberg *et al.*, 2008). Cross-linked products were analysed by immunoblotting.

### Immunoblotting

The detection of fimbrial proteins using specific antisera was performed as described previously (Müller *et al.*, 2006). Primary antisera against H-NS or StpA were derived from immunogenized rabbits and were used in 10 000-fold dilutions. The serum originally directed against StpA strongly reacted with the Hfp protein and therefore could be used for the detection of Hfp. Antiserum recognizing the alpha-subunit of the DNA polymerase was used as a loading control on stripped membranes as performed previously (Müller *et al.*, 2006).

### Capsule ELISA

Quantitative measurements of the production levels of the K15 capsule were performed as described (Schneider *et al.*, 2004).

### *In silico* analyses

Promoter regions were analysed using the bend.it and model.it servers, which are accessible at http://hydra.icgeb.trieste.it/~kristian/dna (Munteanu *et al.*, 1998). Promoter prediction was performed using the BPROM software available at the SoftBerry Homepage (http://www.softberry.com).

### Statistical analyses

Differences between average values were tested for significance by performing an unpaired, two-sided Student's *t*-test. The levels of significance of the resulting *P*-values are reported by the following symbols: * = *P* < 0.05, ** = *P* < 0.01, *** = *P* < 0.001 and n.s. = non-significant.

## References

[b1] Atlung T, Ingmer H (1997). H-NS: a modulator of environmentally regulated gene expression. Mol Microbiol.

[b2] Beloin C, Deighan P, Doyle M, Dorman CJ (2003). *Shigella flexneri* 2a strain 2457T expresses three members of the H-NS-like protein family: characterization of the Sfh protein. Mol Genet Genomics.

[b3] Bertin P, Hommais F, Krin E, Soutourina O, Tendeng C, Derzelle S, Danchin A (2001). H-NS and H-NS-like proteins in Gram-negative bacteria and their multiple role in the regulation of bacterial metabolism. Biochimie.

[b4] Bloch V, Yang Y, Margeat E, Chavanieu A, Auge MT, Robert B (2003). The H-NS dimerization domain defines a new fold contributing to DNA recognition. Nat Struct Biol.

[b5] Carniel E (2001). The *Yersinia* high-pathogenicity island: an iron-uptake island. Microbes Infect.

[b6] Corbett D, Bennett HJ, Askar H, Green J, Roberts IS (2007). SlyA and H-NS regulate transcription of the *Escherichia coli* K5 capsule gene cluster, and expression of *slyA* in *Escherichia coli* is temperature-dependent, positively autoregulated, and independent of H-NS. J Biol Chem.

[b7] Cusick ME, Belfort M (1998). Domain structure and RNA annealing activity of the *Escherichia coli* regulatory protein StpA. Mol Microbiol.

[b8] Dame RT (2005). The role of nucleoid-associated proteins in the organization and compaction of bacterial chromatin. Mol Microbiol.

[b9] Dame RT, Luijsterburg MS, Krin E, Bertin P, Wagner R, Wuite GJL (2005). DNA bridging: a property shared among H-NS-like proteins. J Bacteriol.

[b10] Datsenko KA, Wanner BL (2000). One-step inactivation of chromosomal genes in *Escherichia coli* K-12 using PCR products. Proc Natl Acad Sci USA.

[b11] Deighan P, Beloin C, Dorman CJ (2003). Three-way interactions among the Sfh, StpA and H-NS nucleoid-structuring proteins of *Shigella flexneri* 2a strain 2457T. Mol Microbiol.

[b12] Dobrindt U, Blum-Oehler G, Hartsch T, Gottschalk G, Ron EZ, Fünfstück R, Hacker J (2001). S-fimbria-encoding determinant *sfaI* is located on pathogenicity island III_536_ of uropathogenic *Escherichia coli* strain 536. Infect Immun.

[b13] Dorman CJ (2004). H-NS: a universal regulator for a dynamic genome. Nat Rev Microbiol.

[b14] Doyle M, Dorman CJ (2006). Reciprocal transcriptional and posttranscriptional growth-phase-dependent expression of *sfh*, a gene that encodes a paralogue of the nucleoid-associated protein H-NS. J Bacteriol.

[b15] Doyle M, Fookes M, Ivens A, Mangan MW, Wain J, Dorman CJ (2007). An H-NS-like stealth protein aids horizontal DNA transmission in bacteria. Science.

[b16] Free A, Dorman CJ (1997). The *Escherichia coli stpA* gene is transiently expressed during growth in rich medium and is induced in minimal medium and by stress conditions. J Bacteriol.

[b17] Göransson M, Sonden B, Nilsson P, Dagberg B, Forsman K, Emanuelsson K, Uhlin BE (1990). Transcriptional silencing and thermoregulation of gene expression in *Escherichia coli*. Nature.

[b18] Hommais F, Krin E, Laurent-Winter C, Soutourina O, Malpertuy A, Le Caer JP (2001). Large-scale monitoring of pleiotropic regulation of gene expression by the prokaryotic nucleoid-associated protein, H-NS. Mol Microbiol.

[b19] Johansson J, Uhlin BE (1999). Differential protease-mediated turnover of H-NS and StpA revealed by a mutation altering protein stability and stationary-phase survival of *Escherichia coli*. Proc Natl Acad Sci USA.

[b20] Johansson J, Dagberg B, Richet E, Uhlin BE (1998). H-NS and StpA proteins stimulate expression of the maltose regulon in *Escherichia coli*. J Bacteriol.

[b21] Johansson J, Eriksson S, Sondén B, Wai SN, Uhlin BE (2001). Heteromeric interactions among nucleoid-associated bacterial proteins: localization of StpA-stabilizing regions in H-NS of *Escherichia coli*. J Bacteriol.

[b22] Lang B, Blot N, Bouffartigues E, Buckle M, Geertz M, Gualerzi CO (2007). High-affinity DNA binding sites for H-NS provide a molecular basis for selective silencing within proteobacterial genomes. Nucleic Acids Res.

[b23] Lindberg S, Xia Y, Sondén B, Göransson M, Hacker J, Uhlin BE (2008). Regulatory interactions among adhesin gene systems of uropathogenic *Escherichia coli*. Infect Immun.

[b24] Lithgow JK, Haider F, Roberts IS, Green J (2007). Alternate SlyA and H-NS nucleoprotein complexes control *hlyE* expression in *Escherichia coli* K-12. Mol Microbiol.

[b25] Luijsterburg MS, Noom MC, Wuite GJL, Dame RT (2006). The architectural role of nucleoid-associated proteins in the organization of bacterial chromatin: a molecular perspective. J Struct Biol.

[b26] Madrid C, Balsalobre C, Garcia J, Juarez A (2007). The novel Hha/YmoA family of nucleoid-associated proteins: use of structural mimicry to modulate the activity of the H-NS family of proteins. Mol Microbiol.

[b27] Mayer O, Rajkowitsch L, Lorenz C, Konrat R, Schroeder R (2007). RNA chaperone activity and RNA-binding properties of the *E. coli* protein StpA. Nucleic Acids Res.

[b28] Miller J (1972). Experiments in Molecular Genetics.

[b29] Müller CM, Dobrindt U, Nagy G, Emödy L, Uhlin BE, Hacker J (2006). Role of histone-like proteins H-NS and StpA in expression of virulence determinants of uropathogenic *Escherichia coli*. J Bacteriol.

[b30] Munteanu MG, Vlahovicek K, Parthasarathy S, Simon I, Pongor S (1998). Rod models of DNA: sequence-dependent anisotropic elastic modelling of local bending phenomena. Trends Biochem Sci.

[b31] Owen-Hughes TA, Pavitt GD, Santos DS, Sidebotham JM, Hulton CSJ, Hinton JCD, Higgins CF (1992). The chromatin-associated protein H-NS interacts with curved DNA to influence DNA topology and gene expression. Cell.

[b32] Renzoni D, Esposito D, Pfuhl M, Hinton JCD, Higgins CF, Driscoll PC, Ladbury JE (2001). Structural characterization of the N-terminal oligomerization domain of the bacterial chromatin-structuring protein, H-NS. J Mol Biol.

[b33] Rimsky S, Zuber F, Buckle M, Buc H (2001). A molecular mechanism for the repression of transcription by the H-NS protein. Mol Microbiol.

[b34] Roux A, Beloin C, Ghigo J-M (2005). Combined inactivation and expression strategy to study gene function under physiological conditions: application to identification of new *Escherichia coli* adhesins. J Bacteriol.

[b35] Schneider G, Dobrindt U, Brüggemann H, Nagy G, Janke B, Blum-Oehler G (2004). The pathogenicity island-associated K15 capsule determinant exhibits a novel genetic structure and correlates with virulence in uropathogenic *Escherichia coli* strain 536. Infect Immun.

[b36] Schwyn B, Neilands JB (1987). Universal chemical assay for the detection and determination of siderophores. Anal Biochem.

[b37] Sondén B, Uhlin BE (1996). Coordinated and differential expression of histone-like proteins in *Escherichia coli*: regulation and function of the H-NS analog StpA. EMBO J.

[b38] Sonnenfield JM, Burns CM, Higgins CF, Hinton JC (2001). The nucleoid-associated protein StpA binds curved DNA, has a greater DNA-binding affinity than H-NS and is present in significant levels in *hns* mutants. Biochimie.

[b39] Tendeng C, Bertin P (2003). H-NS in Gram-negative bacteria: a family of multifaceted proteins. Trends Microbiol.

[b40] Travers A, Muskhelishvili G (2005). Bacterial chromatin. Curr Opin Genet Dev.

[b41] Ueguchi C, Seto C, Suzuki T, Mizuno T (1997). Clarification of the dimerization domain and its functional significance for the *Escherichia coli* nucleoid protein H-NS. J Mol Biol.

[b42] Westermark M, Oscarsson J, Mizunoe Y, Urbonaviciene J, Uhlin BE (2000). Silencing and activation of ClyA cytotoxin expression in *Escherichia coli*. J Bacteriol.

[b43] Williams RM, Rimsky S (1997). Molecular aspects of the *E. coli* nucleoid protein, H-NS: a central controller of gene regulatory networks. FEMS Microbiol Lett.

[b44] Yamada H, Muramatsu S, Mizuno T (1990). An *Escherichia coli* protein that preferentially binds to sharply curved DNA. J Biochem.

[b45] Zhang A, Rimsky S, Reaban ME, Buc H, Belfort M (1996). *Escherichia coli* protein analogs StpA and H-NS: regulatory loops, similar and disparate effects on nucleic acid dynamics. EMBO J.

