# Supplementary material for: Differential effects and interactions of endogenous and horizontally acquired H-NS-like proteins in pathogenic Escherichia coli
Source: Mol Microbiol. 2009 Dec 11;75(2):280–93. doi: 10.1111/j.1365-2958.2009.06995.x (PMC2814080; doi:10.1111/j.1365-2958.2009.06995.x)
Supplement: Supplementary file 1 [file mmi0075-0280-SD1.pdf]

Supporting Material

Analysis of Hfp *in vivo* activity (Figure S1)

To test, whether Hfp has any *in vivo* activity, the native protein was expressed from a low copy number plasmid (pCM9) in *hns* mutants of the K-12 strains MG1655 and MC4100, and its ability to complement for the lack of H-NS was assessed for some well described *hns* phenotypes.

As a first test system, *E. coli* K-12 strain MG1655 and its isogenic *hns* mutant BEU740 harbouring plasmid pCM9 or the vector control were stabbed on swarming agar plates to assess their motility. The wt strain with or without the plasmids exhibited swarming ability, whereas the *hns* mutant was completely non-motile. Expression of Hfp from plasmid pCM9, however, restored motility of the *hns* mutant to the same extent as the wt (Fig. S1 A). This indicates that Hfp can assume activation of the flagella master operon *flhCD* in absence of the normal regulator H-NS.

Next, expression of two determinants was analyzed which are usually silent in the wt due to repression by H-NS, namely the cytolysin-encoding gene *clyA* and the *bgl* operon, which encodes enzymes for the utilization of ß-glucosides. Because of transcriptional silencing, *E. coli* K-12 wt cells are non-hemolytic on blood agar plates, and white on agar plates containing the ß-glucosidase substrate X-Glu, whereas *hns* mutants exhibit a hemolytic phenotype and grow as blue colonies on X-Glu plates. When introducing the *hfp*-plasmid into the *hns* mutant, it became non-hemolytic on blood agar and white on X-Glu plates, thereby resembling the wt (Fig. S1 B & C). The same results were obtained with *hns stpA* mutants in an *E. coli* MC4100 background (data not shown). This indicates, that Hfp can silence the *clyA* and *bgl* determinants in the absence of H-NS independently of StpA.

Another typical *hns* phenotype is derepression of the osmotically inducible glycine-betaine transport system encoded by the *proU* operon. Using chromosomal *proU-lacZ* fusion strains in both an hns^{+} (BEU693) and hns^{-} (BEU694) background, a higher ß-glucosidase activity was observed in the *hns* mutant transformed with the vector control than in the wt (1040 vs. 777 Miller units, respectively). After complementation of the mutant with Hfp, *proU* transcription was reduced to wt levels (Fig. S1 D). However, introduction of the *hfp*-containing plasmid into the wild type strain resulted in higher ß-galactosidase activity (1011 Miller units) compared to the vector control strain, thereby indicating, that Hfp might act as an anti-H-NS factor for *proU* repression in this test system.

H-NS was shown to negatively affect its own transcription by autoregulation. To assess, whether expression of the Hfp protein can also mediate repression of *hns* expression, both the *hfp*-encoding plasmid and a plasmid-encoded *hns-lacZ* fusion (pBSN2) were introduced into strain MG1655 and its isogenic *hns* mutant, and ß-galactosidase activity was determined. In the vector control strains, *hns* transcription was 2-fold higher in the *hns* mutant compared to the wt, thereby confirming negative auto-regulation of *hns* expression (Fig. S1 E). This effect was abolished when the mutant was complemented with Hfp, resulting in the same *hns* expression level as in the wt. This result indicates that Hfp is able to repress *hns* transcription independently from H-NS and from other factors specific for UPEC isolates and further confirms a direct transcriptional cross-regulation of the nucleoid-associated proteins.

Stability of Hfp *in vivo* (Figure S2)

The levels of Hfp protein in cells before and after inhibition of protein synthesis were monitored by immunoblot analysis.

Growth studies with 536 mutant derivatives (Figure S3)

The growth ability of the *hfp*, *hns*, and *stpA* mutant series in UPEC strain 536 was assessed on agar plates or in liquid medium at a temperature range between 25°C and 45°C. On agar plates, all H-NS-deficient strains formed smaller colonies than the wt at every temperature (Fig. S3A-C). The most interesting result, however, was obtained when comparing *hns/stpA* to *hns/hfp* double mutants: the *hns/stpA* mutant had a severe growth defect at 45°C (Fig. S3A), whereas the *hns/hfp* mutant still grew at that temperature, though forming very tiny colonies. In liquid medium at 45°C, the generation time of the *hns/stpA* mutant (103 min) was 3.5-times longer than that of the *hns/hfp* mutant (29 min) (Fig. S3A and Table 2). The opposite was true at 25°C, where the *hns/hfp* mutant failed to form colonies on agar plates, while the *hns/stpA* mutant grew forming small colonies (Fig. S3C). These differences in the growth rates of the two double mutants at 25°C were confirmed in liquid medium (Fig. S3C and Table 2), with 1.8-times shorter generation time of the *hns/stpA* mutant (62 min) compared to the *hns/hfp* mutant (111 min).

Analysis of the *hfp* promoter region (Figures S4 & S5)

To further characterize the *hfp* promoter region, various parts of the upstream region of the *hfp* gene were fused to a plasmid-encoded *lacZ* gene and the plasmids were introduced into a *lacZ* mutant derivative of strain 536. Transcriptional activity was measured from both late-log and stationary phase cultures (Fig. S4A). In both growth phases, highest ß-galactosidase activity was measured with a construct encompassing 412 nucleotides upstream of the translational start codon (pCM17). Somewhat lower values were obtained with a shorter fusion of 149 bp in length (pCM16), whereas only background levels were detected in a construct encoding 69 nucleotides of the upstream region (pCM15). Interestingly, the transcriptional activity observed with the constructs pCM18 and pCM19, containing 746 bp and 1091 bp of the upstream region, respectively, was considerably lower compared to the 412-bp construct in all cases (Fig. S4A). Analysis of the nucleotide composition of the various promoter fragments revealed that the additional nucleotide sequences present in pCM18 and pCM19 contains several AT-rich stretches, which commonly results in a high degree of predicted DNA curvature (Fig. S4B). Therefore, the additional sequence in the far upstream region affecting the *hfp* locus might be a preferential target for H-NS family proteins, resulting in a higher degree of repression of *hfp* expression. Moreover, the transcriptional start site of *hfp* identified by primer extension analysis (Fig. S5) lies within the coding sequence of a putative open reading frame (encoding the hypothetical protein ECP_1926) reading into the same direction as *hfp*. Therefore, the decrease in ß-galactosidase activity observed with plasmids pCM18 and pCM19 (Fig. S4A) might also be caused by transcriptional interference.

REFERENCES

Berger, H., J. Hacker, A. Juarez, C. Hughes & W. Goebel, (1982) Cloning of the chromosomal determinants encoding hemolysin production and mannose-resistant hemagglutination in *Escherichia coli*. *J. Bacteriol.* **152**: 1241-1247.

Casadaban, M. J., (1976) Transposition and fusion of the *lac* genes to selected promoters in *Escherichia coli* using bacteriophage lambda and Mu. *J. Mol. Biol.* **104**: 541-555.

Dobrindt, U. & J. Hacker, (2001) Regulation of the tRNA5Leu-encoding gene *leuX* that is associated with a pathogenicity island in the uropathogenic *Escherichia coli* strain 536. *Mol. Genet. Genom.* **265**: 895-904.

Espinosa-Urgel, M., C. Chamizo & A. Tormo, (1996) A consensus structure for sigma S-dependent promoters. *Mol. Microbiol.* **21**: 657-659.

Free, A. & C. J. Dorman, (1997) The *Escherichia coli stpA* gene is transiently expressed during growth in rich medium and is induced in minimal medium and by stress conditions. *J. Bacteriol.* **179**: 909-918.

Kalogeraki, V. S. & S. C. Winans, (1997) Suicide plasmids containing promoterless reporter genes can simultaneously disrupt and create fusions to target genes of diverse bacteria. *Gene* **188**: 69-75.

Lang, B., N. Blot, E. Bouffartigues, M. Buckle, M. Geertz, C. O. Gualerzi, R. Mavathur, G. Muskhelishvili, C. L. Pon, S. Rimsky, S. Stella, M. Madan Babu & A. Travers, (2007) High-affinity DNA binding sites for H-NS provide a molecular basis for selective silencing within proteobacterial genomes. *Nucl. Acids Res.* **35**: 6330–6337.

Müller, C. M., U. Dobrindt, G. Nagy, L. Emödy, B. E. Uhlin & J. Hacker, (2006) Role of histone-like proteins H-NS and StpA in expression of virulence determinants of uropathogenic *Escherichia coli*. *J. Bacteriol.* **188**: 5428-5438.

Nagarajavel, V., S. Madhusudan, S. Dole, A. R. Rahmouni & K. Schnetz, (2007) Repression by binding of H-NS within the transcription unit. *J. Biol. Chem.* **282**: 23622-23630.

Reznikoff, W. & W. McClure, (1986) *E. coli* promoters. In Maximizing gene expression. Reznikoff, W., and Gold, L. (eds). Boston, MA: Butterworths. 1-33.

Schneider, G., U. Dobrindt, H. Brüggemann, G. Nagy, B. Janke, G. Blum-Oehler, C. Buchrieser, G. Gottschalk, L. Emödy & J. Hacker, (2004) The pathogenicity island-associated K15 capsule determinant exhibits a novel genetic structure and correlates with virulence in uropathogenic *Escherichia coli* strain 536. *Infect. Immun.* **72**: 5993-6001.

Skerra, A., (1994) Use of the tetracycline promoter for the tightly regulated production of a murine antibody fragment in *Escherichia coli*. *Gene* **151**: 131-135.

Sondén, B. & B. E. Uhlin, (1996) Coordinated and differential expression of histone-like proteins in *Escherichia coli*: regulation and function of the H-NS analog StpA. *EMBO J.* **15**: 4970-4980.

Wang, R. F. & S. R. Kushner, (1991) Construction of versatile low-copy-number vectors for cloning, sequencing and gene expression in *Escherichia coli*. *Gene* **100**: 195-159.

Wolf, T., W. Janzen, C. Blum & K. Schnetz, (2006) Differential dependence of StpA on H-NS in autoregulation of *stpA* and in regulation of *bgl*. *J. Bacteriol.* **188**: 6728-6738.

Zhang, A., S. Rimsky, M. E. Reaban, H. Buc & M. Belfort, (1996) *Escherichia coli* protein analogs StpA and H-NS: regulatory loops, similar and disparate effects on nucleic acid dynamics. *EMBO J.* **15**: 1340-1349.

SUPPORTING TABLES

Table S1. Comparison of *stpA* and *hfp* expression.

	stpA	*hfp*
Growth Phase-dependent expression	transiently expressed during mid-log phase [a]	induced upon entry into stationary phase
Promoter activity in mid-log phase (37°C)	504 Miller Units [b] (332-bp fragment)	41 Miller Units (412-bp fragment; Fig. S5A)
Promoter activity in stationary phase (37°C)	79 Miller Units [c] (517-bp fragment)	256 Miller Units (412-bp fragment; Fig. S5A)
Temperature-dependent expression	2x higher at 37°C than at 26°C [d] (log phase)	10x higher at 25°C than at 37°C (stationary phase; Fig. 3A)
Repression by H-NS	+ (3 to 10-fold at 37°C) [e]	+ (4-fold at 30°C (Fig. 3B&D); 12-fold at 37°C (data not shown))
Repression by StpA	+ (3 to 6-fold) [f]	- (as indicated in Fig. 3D)
Repression by Hfp	- (as suggested in Fig. 4A)	+ (as suggested in Fig. 4A)
Regulation by Lrp	+ (2x higher in minimal medium) [g]	- (data not shown)
Regulation by Fis	- [h]	+ (1.7-fold activation in absence of H-NS (data not shown))

Comment: [a] determined by Northern blot analysis (Free & Dorman, 1997)
[b] measured using *lacZ*-promoter fusions cloned into pRZ5202 (Sondén & Uhlin, 1996)
[d] measured using *lacZ*-promoter fusions cloned into pQF50 (Free & Dorman, 1997)
[d] determined by Northern blot analysis (Sondén & Uhlin, 1996)
[e] determined by Northern blot analysis (Sondén & Uhlin, 1996, Free & Dorman, 1997) or using *lacZ* fusion constructs (Wolf *et al.*, 2006)
[f] measured using chromosomal *lacZ* fusion constructs (Wolf et al., 2006)
[g] determined by Northern blot analysis (Sondén & Uhlin, 1996) or measured using *lacZ*-promoter fusions cloned into pQF50 (Free & Dorman, 1997)
[h] assessed by Northern blot analysis (Free & Dorman, 1997)

Table S2. Bacterial strains and plasmids used in this study.

Strain or Plasmid	Relevant Characteristics	Reference or Source
Bacterial strain		
UPEC 536	serotype O6:K15:H31; Sm^r	Berger *et al.* (1982)
536 *ΔlacZ*	*lacZ*$^-$	Dobrindt & Hacker (2001)
536 *ΔlacZ Δhns::cat*	*hns* gene replaced with *cat* cassette in *lacZ* strain	This study
536*Δhfp*	*hfp* inactivated by complete deletion	This study
536*Δhns*	*hns* inactivated by complete deletion	Müller *et al.* (2006)
536*ΔstpA*	*stpA* inactivated by complete deletion	Müller *et al.* (2006)
536*ΔhnsΔstpA*	*hns* and *stpA* inactivated by complete deletion	Müller *et al.* (2006)
536*ΔhfpΔstpA*	*hfp* and *stpA* inactivated by complete deletion	This study
536*ΔhfpΔhns*	*hfp* and *hns* inactivated by complete deletion	This study
536*ΔhfpΔhnsΔstpA*	*hfp, hns,* and *stpA* inactivated by complete deletion	This study
536 ΔK15	deletion of the complete K15 *kps* locus, Cm^r	Schneider *et al.* (2004)
536 *ΔlacZ hfp::lacZYA::neo*	chromosomal *lacZ*-transcriptional fusion; Km^r	This study
536 *ΔlacZ hfp::lacZYA::neo Δhns::cat*	*hns* gene replaced with *cat* cassette in chromosomal *lacZ* fusion strain	This study
536 *ΔlacZ hfp::lacZYA::neo ΔstpA::cat*	*stpA* gene replaced with *cat* cassette in chromosomal *lacZ* fusion strain	This study
DH5α	F$^-$, *end*A1, *hsd*R17 (r_k^-, m_k^-), *sup*E44, *thi*-1, *rec*A1, *GyrA*96, *rel*A1, λ$^-$, Δ(*argF-lac*)U169, Φ80*dlacZ* ΔM15	BRL, Bethesda Research Laboratories
E. coli Rosetta^{TM} (DE3)	F$^-$ *ompT hsdS gal dcm lacY*1 (DE3) pRARE (Cm^R)	Merck Biosciences
BL21(DE3) *Δhns*	*hns::*Km^r in BL21 (*hsdS gal* (λcI*ts*857 *ind*-1 *Sam*7 *nin*-5 *lacUV5*-T7 gene 1)	Zhang *et al.* (1996)
BEU693	GM37, *trp*::Tn 10. *proU-lac Z*	Sondén & Uhlin (1996)
BEU694	BEU693 *Δhns*	Sondén & Uhlin (1996)
MC4100	F- *araD139 Δ(argF-lac)U169 rpsL150* (Strr) *relA1 flbB5301 deoC1 ptsF25 rbsR*	Casadaban (1976)
CMM4100	*hns* and *stpA* inactivated by complete deletion in MC4100	This study
CMM4101	*lon* inactivated by complete deletion in CMM4101	This study
Plasmids		
pASK75	*tet* $^{p/o}$, *bla/tetR* transcriptional fusion vector	Skerra (1994)
pCM8	sequence encoding His_6-tagged Hfp protein inserted into the aTc-inducible expression vector pASK75	This study
pWKS30	low copy number cloning vector; *bla*	Wang & Kushner (1991)
pCM9	*hfp* gene and 200 bases up- and downstream cloned into pWKS30	This study
pRZ5202	transcriptional *lacZ* fusion vector, Cb^r	Reznikoff & McClure (1986)
pCM15	transcriptional fusion of the *hfp* promoter region (69 bp) to *lacZ* in pRZ5202	This study
pCM16	transcriptional fusion of the *hfp* promoter region (149 bp) to *lacZ* in pRZ5202	This study
pCM17	transcriptional fusion of the *hfp* promoter region (412 bp) to *lacZ* in pRZ5202	This study

Table S2 - continued		
pCM18	transcriptional fusion of the *hfp* promoter region (746 bp) to *lacZ* in pRZ5202	This study
pCM19	transcriptional fusion of the *hfp* promoter region (1091 bp) to *lacZ* in pRZ5202	This study
pVIK112	*lacZYA* transcriptional fusion vector, Km^r	Kalogeraki & Winans (1997)
pBSN2	translational fusion *hns* in pMW156, Km^r	Sondén & Uhlin (1996)
pKENV61	BglG-independent transcriptional *bgl::lacZ* fusion on pACYC backbone (p*bglG*-RATstab/t1-*gblG*-*lacZ attP aadA*); Km^r	Nagarajavel *et al*. (2007)
pKENV61*Δ*	EcoRV-restricted and religated pKENV61	This study

Table S3. Oligonucleotides used in this study.

Name	Sequence (5'-3')
Mutagenesis	
hfp-1	GCA TT A TTT TTA TTA ATC GCC TAT TTT AAA ACA CCG GAT GAC ATA CTA TGG TGT AAG CTG GAG CTG CTT
hfp-2	CTG TTA TCT TGA GAA CAG TGA AGC CCG GAT ATG CTG TAG CGG TTA CAT ATG AAT ATC CTC CTT AGT TCC TA
hns-1	AAA TCC CGC CGC TGG CGG GAT TTT AAG CAA GTG CAA TCT ACA AAA GAT TAG TGT AGG CTG GAG CTG CTT
hns-2	ATT ATT ACC TCA ACA AAC CAC CCC AAT ATA AGT TTG AGA TTA CTA CAA TGC ATA TGA ATA TCC TCC TTA GTT CCT A
stpA-1	AGA AGC GAC GCC GGA CGC GCC CTA GCA GCG ACA TCC GGC CTC AGT AAT TAG TGT AGG CTG GAG CTG CTT
stpA-2	GCG ACG AAA TAC TTT TTT TGT TTT GGC GTT AAA AGG TTT TCT TTA TTA TGC ATA TGA ATA TCC TCC TTA GTT CCT
lon-1	GTC ATC TGA TTA CCT GGC GGA AAT TAA ACT AAG AGA GAG CTC TAT GGT GTA GGC TGG AGC TGC T
lon-2	CCT GCC AGC CCT GTT TTT ATT AGT GCA TTT TGC GCG AGG TCA CTA CAT ATG AAT ATC CTC CTT AGT TCC TA
rpoS-1	GGC TTT TGC TTG AAT GTT CCG TCA AGG GAT CAC GGG TAG GAG CCA CCT TAT GGT GTA GGC TGG AGC TGC TT
rpoS-2	GGC CAG CCT CGC TTG AGA CTG GCC TTT CTG ACA GAT GCT TAC TTA CAT ATG AAT ATC CTC CTT TAG TTC CTA
fis-1	CTT CGA AAA TTT TGC GTA AAC AGA AAT AAA GAG CTG ACA GAA CTA TGG TGT AGG CTG GAG CTG CTT
fis-2	GCC GAG TAG CGC CTT TTT AAT CAA GCA TTT AGC TAA CCT GAA TTA CAT ATG AAT ATC CTC CTT TAG TTC CTA
hfp-lacZ1	GCT ATC GAA GCT GGT AAG ACA CTG GAA GAC TTT GCT ATC TAA CCG CTA CAG CAT TAG ATG ACT GAC AGG AAA CAG CTA TG
hfp-lacZ2	GGA TGA ATA ATC GAA TAT TTT CCG GGC CTG TTA TCT TGA GAA CAG TGA CCC GGG GTG GGC GAA GAA CTC CAG
Plasmid construction (restriction sites are underlined, translational start and stop codons are in bold)	
Hfp-fw-HindIII	GCG AAG CCT CCA TCA AAC CAA AAG
Hfp-rv-XbaI	GCG TCT AGA GCT GCG TAT CAT TGG TGG
XhoI-His-hfp	TGC TCT AGA **TAG TAA** AGG AAA AAC T **ATG** CAT CAC CAT CAC CAT CAC *ATC GAG GGC AGG* **ATG** AGT GAA GCT CTT AAG GCA CTG
Hfp-HindIII	TGC AAG CTT **TTA** GAT AGC AAA GTC TTC CAG
Hfp-BamHI-rv	GCG GGA TCC CAG TGC CTT AAG AGC TTC AC
Hfp-EcoRI-1	GCG GAA TTC GAT TTT TCC AAC AGC ACC GT
Hfp-EcoRI-344	GCG GAA TTC CCC ATC AGC AGT TCA AAA GC
Hfp-EcoRI-679	GCG GAA TTC GGC GAG ACA AAT CAA CAG A
Reverse-Transcription-PCR	
hfp-RT1	CGT ACA CTT CGC GCA CAG GC
hfp-RT2	CCA GTG TCT TAC CAG CTT CG
hns-RT1	CAG CTG GAG TAC GGC CTT GG
hns-RT2	CGT ACT CTT CGT GCG CAG GC
stpA-RT1	GCA ATT GGC TTC GGT GTA CG
stpA-RT2	TCG CGA ATT CTC CAT TGA CG
16S-1	TCT CCT GAG AAC TCC GGC A
16S-2	CAG CGT TCA ATC TGA GCC A
Primer Extension / Sequencing	
hfp-primext	CAG TGC CTT AAG AGC TTC AC
hfp-seq-fw	CGG TAA TGA GCA GAC TTG GAC G
lacZ-seq-2 -rv	GCC ATT CGC CAT TCA GGC TGC
EMSA promoter fragments	
hfp-prom1	GCG ATG AAG AAA TAG TAG CC
hfp-prom2	CGC GAA GTG TAC GAA TAT TG
hns-prom1	CCC TGC TCT TAT TGC GAC TG
hns-prom2	CAC GAA GAG TAC GGA TGT TG
stpA-prom1	GAC AGC AGA AAG CAC CAG TG
stpA-prom2	ACG GAG GGT GCG AAT GTT AT

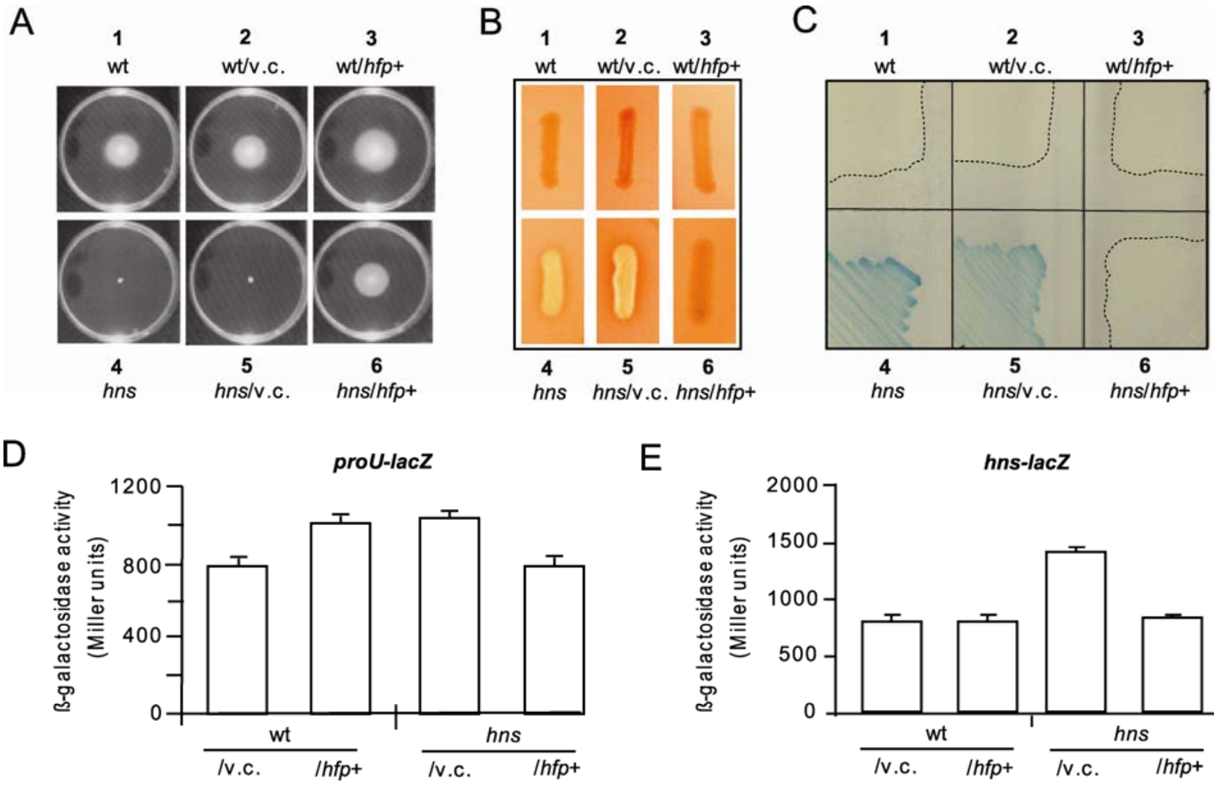

Fig. S1. Complementation ability of Hfp for the lack of H-NS in *E. coli* K-12 strains. *E. coli* strains MG1655 or MC4100 and isogenic *hns* mutants were transformed with low copy number plasmid pCM9 encoding the *hfp* gene with its native promoter, or the vector control (pWKS30), and changes in typical *hns* phenotypes were monitored as described in the Experimental procedures section in the main article. Numbers in panels A to C represent the following strains: 1 = wt (MG1655); 2 = wt /pWKS30; 3 = wt /pCM9; 4 = *hns* (BEU740); 5 = *hns* /v.c.; 6 = *hns* /pCM9. (A) Swarming ability on 0.3 % agar plates assessed after 16 h incubation; (B) Haemolytic activity on blood agar plates; (C) Expression of ß-glucosidases (*bgl*) on X-Glu plates (the outline of the white colony streak is marked for better visualisation); (D) ß-galactosidase activity of chromosomal *proU-lacZ* reporter strains reporting expression of the osmotically inducible glycine-betaine transport system. Strains BEU693 (wt) and BEU694 (*hns*) transformed with either pCM9 (/hfp^{+}) or the vector control (/v.c.) were grown at 37°C in minimal medium A supplemented with 0.3 M NaCl in order to optimise the expression of the *proU* gene as described previously (Sondén & Uhlin, 1996); (E) Transcriptional activity of a plasmid-encoded *hns-lacZ* reporter construct (pBSN2). Strains MG1655 (hns^{+}) and BEU740 (hns^{-}) were subsequently transformed with pBSN2 and either pCM9 (/hfp^{+}) or the vector control (/v.c.), and *hns* expression was monitored by measuring ß-galactosidase activity from mid-log phase cultures grown at 37°C. In both D and E the mean values and standard deviations from three independent experiments are shown.

Fig. S1

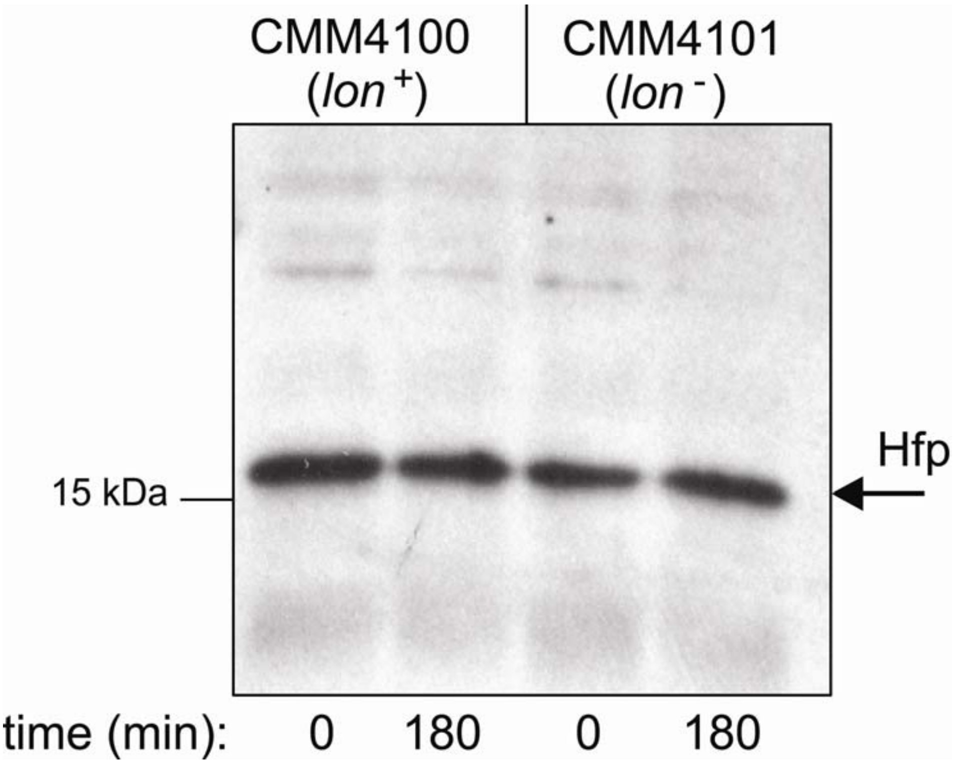

Fig. S2. Analysis of Hfp stability. Immunoblot analysis for the detection of Hfp protein before and 3 h after inhibition of protein synthesis by addition of spectinomycin (see Experimental procedures of main article). Strains used were the MC4100 derivatives CMM4100 (*ΔhnsΔstpA*) and CMM4101 (*ΔhnsΔstpAΔlon*), both expressing *hfp* from plasmid pCM9.

Fig. S2

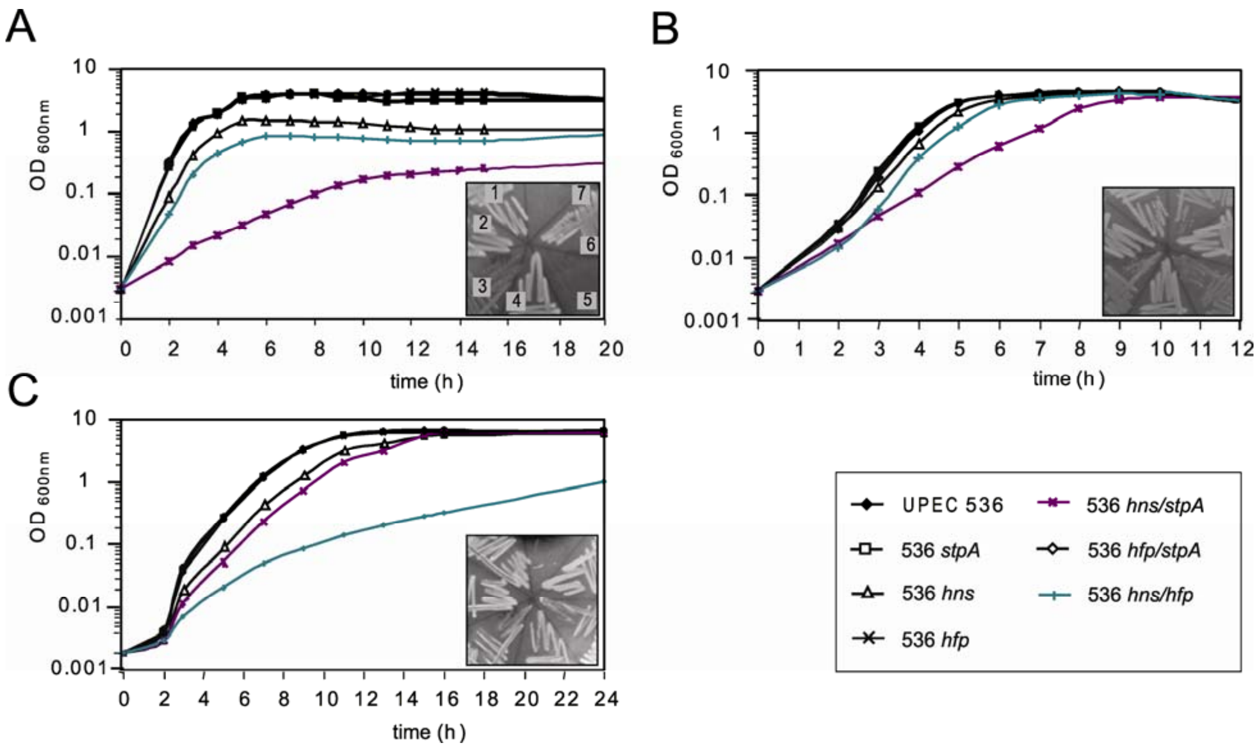

Fig. S3. Effect of the lack of nucleoid proteins on growth at different temperatures. Growth curves of UPEC strain 536 and isogenic mutants grown at 45°C (A), 37°C (B), or 25°C (C) in 20 ml LB broth with aeration. The inserts show pictures of the same strains grown on agar plates at each temperature. The order of the strains was the same on each plate and is indicated by numbers in panel A, where 1 = 536 wt, 2 = Δ*stpA*, 3 = Δ*hns*, 4 = Δ*hfp*, 5 = Δ*stpA*/Δ*hns*, 6 = Δ*stpA*/Δ*hfp*, and 7 = Δ*hns*/Δ*hfp*.

Fig. S3

A

Fusion plasmid	*hfp-lacZ*	ß-galactosidase activity (Miller Units) wt - late-log	wt - stationary	*hns* - stationary
pCM19	(-1091)	18 ± 0.1	90 ± 4.2	406 ± 12.7
pCM18	(-746)	9 ± 0.3	62 ± 4.3	479 ± 15.1
pCM17	(-412)	41 ± 2.9	256 ± 7.8	886 ± 1.2
pCM16	(-149)	27 ± 0.8	214 ± 13.5	725 ± 3.1
pCM15	(-69)	1 ± 0.1	12 ± 0.2	52 ± 3.1
pRZ5202	(v.c.)	1 ± 0.1	8 ± 0.6	43 ± 0.6

B

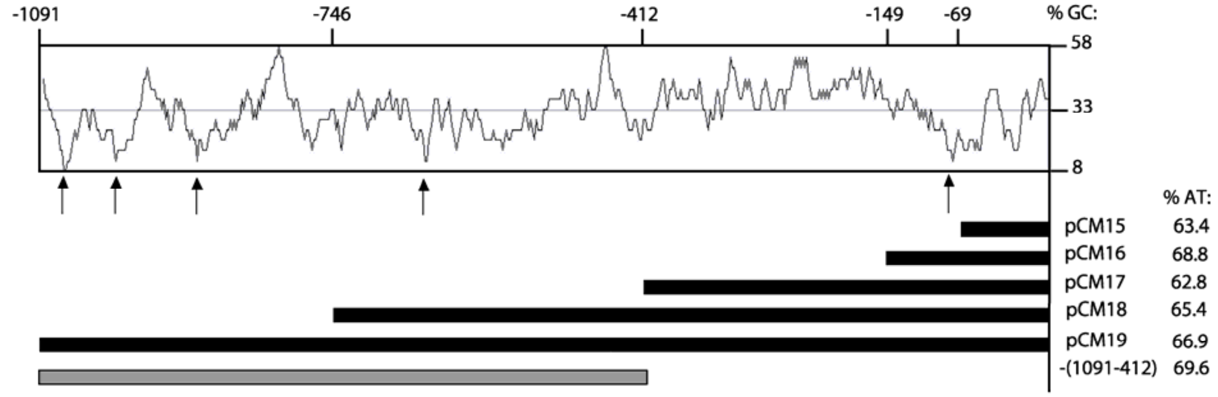

Fig. S4. Analysis of the *hfp* upstream region. (A) Transcriptional activity obtained from plasmid constructs (plasmids pCM15-pCM19) containing different lengths of the *hfp* upstream region fused to a promoter-less *lacZ* gene of plasmid pRZ5202 in strain 536 Δ*lacZ* and isogenic *hns* mutant derivative. The strains were grown at 37°C in LB medium. Samples were taken at late-log phase (OD=1) or in stationary phase (OD=3) and ß-galactosidase activity was measured. Values represent means and SD of three independent experiments. (B) GC-content of the region spanning 1091 nucleotides upstream of the translational start codon of the *hfp* gene (upper panel). The regions covered by the transcriptional *lacZ* fusion plasmids, pCM15 to pCM19, are indicated by back bars in the lower half of the figure. The calculated AT-content over the entire length of the insert of each plasmid is presented on the right hand side. Regions with unusual low GC-content are highlighted by black arrows. The AT-content of the additional nucleotide sequence (spanning the region from –1091 to –412; gray bar) present in plasmids pCM18 and pCM19 only is also indicated in the figure.

Fig. S4

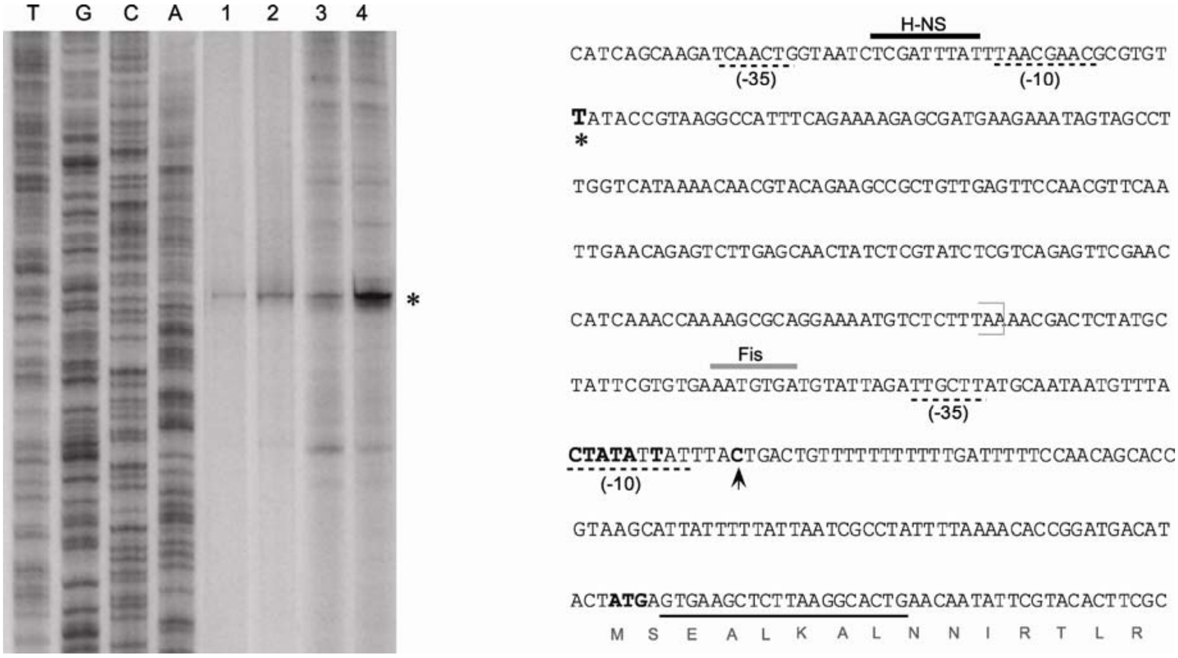

Fig. S5: Analysis of the *hfp* promoter and transcriptional start by primer extension. RNA was extracted from cultures of wild-type UPEC strain 536 and an isogenic *hns* mutant grown at 30°C for 16 h. The binding site for the ^{32}P end-labelled oligonucleotide (Hfp-primext) is underlined in the sequence shown in the right panel. The sequence ladder was derived from DNA of plasmid pCM20 using the same primer. An asterisk indicates the major extension product (left panel) and the corresponding transcription initiation site within the upstream sequence (right panel). A possible second transcription initiation site is indicated by the vertical arrowhead below the sequence. The putative –10 and –35 boxes predicted by the BPROM software are indicated. The stop codon for translation of the putative upstream ORF, ECP_1926, is indicated by a grey bracket. The *rpoS* consensus sequence (Espinosa-Urgel *et al.*, 1996) and the translation initiation codon are marked with bold lettering. Lanes: 1 = 536 wt (5 µg RNA); 2 = 536 *hns* (5 µg RNA); 3 = 536 wt (40 µg RNA); 4 = 536 *hns* (40 µg RNA). Predicted Fis (score –11, BPROM analysis) and H-NS binding sites (alignment with H-NS consensus sequences proposed by Lang *et al.* (2007)) are marked by grey and black lines above the sequence, respectively.

Fig. S5
